# Overweight People Have *Low* Levels of Implicit Weight Bias, but Overweight Nations Have *High* Levels of Implicit Weight Bias

**DOI:** 10.1371/journal.pone.0083543

**Published:** 2013-12-17

**Authors:** Maddalena Marini, Natarajan Sriram, Konrad Schnabel, Norbert Maliszewski, Thierry Devos, Bo Ekehammar, Reinout Wiers, Cai HuaJian, Mónika Somogyi, Kimihiro Shiomura, Simone Schnall, Félix Neto, Yoav Bar-Anan, Michelangelo Vianello, Alfonso Ayala, Gabriel Dorantes, Jaihyun Park, Selin Kesebir, Antonio Pereira, Bogdan Tulbure, Tuulia Ortner, Irena Stepanikova, Anthony G. Greenwald, Brian A. Nosek

**Affiliations:** 1 Department of Psychology, University of Virginia, Charlottesville, Virginia, United States of America; 2 Department of Communication and Economics, University of Modena and Reggio Emilia, Reggio Emilia, Italy; 3 Department of Psychology, University of Potsdam, Potsdam, Germany; 4 Department of Psychology, Warsaw University, Warsaw, Poland; 5 Department of Psychology, San Diego State University, San Diego, California, United States of America; 6 Department of Psychology, Stockholm University, Stockholm, Sweden; 7 Department of Psychology, University of Amsterdam, Amsterdam, The Netherlands; 8 Institute of Psychology, Chinese Academy of Sciences, Beijing, China; 9 Department of Psychology, Eötvös Loránd University, Budapest, Hungary; 10 Ferris University, Yokohama, Japan; 11 Department of Psychology, University of Cambridge, Cambridge, United Kingdom; 12 Faculdade de Psicologia e de Ciências da Educação, Universidade do Porto, Porto, Portugal; 13 Department of Psychology, Ben-Gurion University of the Negev, Beer Sheva, Israel; 14 Department of Applied Psychology, University of Padova, Padova, Italy; 15 Instituto Electoral Veracruzano, Xalapa, Mexico; 16 Facultad de Psicología, Universidad Autónoma del Estado de Morelos, Cuernavaca, Morelos, México; 17 Department of Psychology, Baruch College-City University of New York, New York, NY; 18 London Business School, Regent's Park, London, UK; 19 Brain Institute Federal, University of Rio Grande do Norte, Natal, Rio Grande do Norte, Brazil; 20 Department of Psychology, Transilvania University of Brasov, Brasov, Romania; 21 Department of Psychology, Division of Psychological Assessment, University of Salzburg, Salzburg, Austria; 22 University of South Carolina, Sloan College, Columbia, South Carolina, United States of America; 23 Department of Psychology, University of Washington, Seattle, Washington, United States of America; University of Bologna, Italy

## Abstract

Although a greater degree of personal obesity is associated with *weaker* negativity toward overweight people on both explicit (i.e., self-report) and implicit (i.e., indirect behavioral) measures, overweight people still prefer thin people on average. We investigated whether the national and cultural context – particularly the national prevalence of obesity – predicts attitudes toward overweight people independent of personal identity and weight status. Data were collected from a total sample of 338,121 citizens from 71 nations in 22 different languages on the Project Implicit website (https://implicit.harvard.edu/) between May 2006 and October 2010. We investigated the relationship of the explicit and implicit weight bias with the obesity both at the individual (i.e., across individuals) and national (i.e., across nations) level. Explicit weight bias was assessed with self-reported preference between overweight and thin people; implicit weight bias was measured with the Implicit Association Test (IAT). The national estimates of explicit and implicit weight bias were obtained by averaging the individual scores for each nation. Obesity at the individual level was defined as Body Mass Index (BMI) scores, whereas obesity at the national level was defined as three national weight indicators (national BMI, national percentage of overweight and underweight people) obtained from publicly available databases. Across individuals, greater degree of obesity was associated with *weaker* implicit negativity toward overweight people compared to thin people. Across nations, in contrast, a greater degree of national obesity was associated with *stronger* implicit negativity toward overweight people compared to thin people. This result indicates a different relationship between obesity and implicit weight bias at the individual and national levels.

## Introduction

Overweight and obese individuals face prejudice and discrimination in many areas of daily life [1,2]. This stigmatization threatens overweight and obese individuals' psychological and physical health by increasing the risk for depression, anxiety, low self-esteem, and body dissatisfaction [3,4]. The psychological toll of weight stigmatization can be devastating. Overweight youth who are stigmatized about their weight are 2-3 times more likely to engage in suicidal thoughts and behaviors compared with their overweight peers who are not stigmatized [5]. Obese individuals who experience weight stigma are more likely to engage in unhealthy eating behaviors and lower levels of physical activity [6,7] and are less likely to undergo age-appropriate preventive cancer screenings [8,9]. Indeed, negativity toward overweight and obese people is also shown in the health care setting by physicians [10], nurses, medical students, dietitians and psychologists. Similarly, coworkers, employers, teachers, peers, friends, family members and romantic partners show weight bias [1,2].

Weight bias has been documented both explicitly [11,12], through consciously held evaluations which are potentially subject to social desirability and rely on self-report, and implicitly [13–17], through automatic evaluations that occur outside of awareness or conscious control. Some reasons for the existence of weight bias include valuation of a thin ideal for beauty, stereotypes such as laziness associated with obesity, and perceptions of the controllability of weight [18]. However, both explicit and implicit measures have highlighted the pervasiveness of weight bias by showing that, although overweight and obese people possess less weight bias than normal weight and underweight people, they still prefer thin people on average [19]. This suggests that overweight and obese people might find it difficult to identify with and prefer their weight group because of weight stigmatization [20]. Thus, factors other than the individual's identity - which promotes strong tendencies to prefer one’s own groups and social characteristics [21] - might be shaping weight bias. 

One of those factors could be the cultural context. Culture powerfully shapes the beliefs and behavior of its members [22]. Existing studies show some variations in weight bias across nations [23]. For example, opposing the overall trend, in some nations popular stereotypes associate obesity with more positive than negative characteristics [24–27]. Overweight and obese people’s persisting negativity toward their own group could be partly influenced by negativity toward overweight people at the national level.

For example, health and beauty are conventionally associated with thinness. Thus, in nations with a high prevalence of overweight and obese people, the starker contrast of models of health and beauty with the general population may amplify the desirability and social status of the thin ideal. This could increase cultural messages, advertising, and focus on the desirability of thinness relative to obesity. 

Likewise, the health consequences of obesity are well understood. Obesity increases the likelihood of various diseases, particularly heart disease, type 2 diabetes, obstructive sleep apnea, certain types of cancer, and osteoarthritis [28] and ^,^the risk of many physical conditions such as high blood pressure, high blood cholesterol and high triglyceride levels [29]. In nations with a high incidence of overweight people, the public discussion health concerns and consequences might lead to an increase in negativity towards obesity even though more people are obese.

These likely cultural influences suggest the possibility that the relationship between weight bias and obesity across nations could be exactly the opposite of what observed at the individual level. For an overweight or obese individual, the evaluation of oneself, one’s groups, and one’s characteristics predicts greater *positivity* toward obesity than a normal or underweight individual. For a nation with a high prevalence of overweight and obese people, the negative social and health consequences of obesity may predict greater *negativity* toward obesity than a nation with low prevalence of overweight people.

To investigate the potential influence of obesity on the weight bias at the national level, we assessed the explicit (i.e., self-reported) and implicit (i.e., automatic or non-conscious) weight bias of citizens from 71 nations and investigated their relationship with the obesity comparing across individuals and nations.

## Materials and Methods

### Ethics Statement

The present research was approved by the University of Virginia Institutional Review Board for the Social and Behavioral Sciences.

### Procedure

Data were collected among volunteers at the Project Implicit website (https://implicit.harvard.edu) between May 2006 and October 2010. Project Implicit offers visitors an opportunity to participate in research and receive educational feedback on a variety of social attitudes and stereotypes. Participants selected the weight study from among a list of options. The study was available in the following 22 languages: Bosnian, Chinese, Czech, Dutch, English, Flemish, French, German, Hebrew, Hungarian, Italian, Japanese, Korean, Norwegian, Polish, Portuguese, Romanian, Russian, Spanish, Swedish, Thai and Turkish. 

Stimuli used in the study were translated from English to different languages and validated by native speakers. The weight study included a demographic questionnaire, a questionnaire about weight attitudes and beliefs, and an Implicit Association Test (IAT) presented in a randomized order [30]. All together, the study required about 10 minutes to complete.

### Participants

A sample of 338,121 individuals from 71 nations met our inclusion criteria: (a) reported a country of citizenship, (b) came from a country of citizenship that was represented in the compiled national indicators, and (c) that country had at least 100 study sessions from which to calculate weight bias. Mean sample size by country was 4,762 (SD=26,842) with the smallest samples being Costa Rica (N=104), Egypt (N=106), and Lebanon (N=108) and the largest being the United States (N=226,613). 70.5% of those reporting gender were female, and the mean age was 27.8 (SD=10.64; age=18-89). For the convenience of analyses and presentation, we list some territories as nations. A breakdown by nation of the demographic and descriptive statistics from Project Implicit website appears in Table S1 in File S1.

### Measures

#### Implicit Association Test

The IAT measured association strengths between the concepts *thin* and *fat* and the attributes *good* and *bad*. The IAT has been used in hundreds of published research studies assessing social preferences and has amassed a large literature clarifying its extraneous influences, and construct and predictive validity [31–36]. In particular, several previous studies have shown that the IAT is an effective experimental tool to assess weight bias [14,19,34].

The IAT procedure followed the standard described by Nosek, Greenwald, and Banaji [37], and used the stimuli reported by Nosek et al. [34]. Participants categorized pictures or words representing the four categories – thin, fat, good, bad - in two different sorting conditions. Stimuli representing the four categories were presented one at a time in the center of the computer screen, and participants categorized each of them by pressing one of two keys. In one condition, participants categorized pictures representing thin people and good words (i.e., joy, love, peace, wonderful, pleasure, glorious, laughter and happy) with one response key, while categorizing pictures representing overweight people and bad words (i.e., agony, terrible, horrible, nasty, evil, awful, failure and hurt) by using another response key. In the other condition, participants categorized the same pictures and words but with a different key configuration: this time pictures of thin people and bad words were categorized with one key whereas pictures of overweight people and good words with the other. The order of these conditions was randomized across participants.

The difference in average categorization latency between the two conditions is an indicator of association strengths between the weight and evaluative categories. For example, faster categorization when thin people and good shared a response key (and overweight people and bad shared a response key) compared to the reverse indicated an implicit preference for thin people compared to overweight people. Following Greenwald, Nosek and Banaji [38], IAT scores were computed for each participant by dividing the difference in mean response latency between the two IAT conditions by the participant’s latency standard deviation inclusive of the two conditions. The IAT score could range from +2 to -2, with zero indicating no relative preference between thin people and overweight people. More positive scores indicated stronger associations of thin people with good and overweight people with bad compared to associations of thin people with bad and overweight people with good, and they were interpreted as an implicit preference for thin people over overweight people. Conversely, more negative scores indicated stronger associations of thin people with bad and overweight people with good compared to associations of thin people with good and overweight people with bad, and they were interpreted as an implicit preference for overweight people over thin people.

We analyzed our IAT data in two steps following procedures described and validated in the literature [17,38]. In the first step we computed the IAT scores according to the improved scoring algorithm described by Greenwald, Nosek. and Banaji [38] with the following features: responses faster than 350 milliseconds were removed, responses slower than 10,000 milliseconds were removed, and errors were replaced with the mean of the correct responses in that response block plus a 600 millisecond penalty [39]. In the second step we removed from our data set subjects whose results were indicative of careless participation. In particular, we removed IAT sessions a priori for: (1) going too fast (< 350 ms) on more than 10% of the total test trials, (2) making more than 30% erroneous responses across the critical blocks (see also Nosek et al. [17]) 10.15% of IATs were removed based on these exclusion criteria.

#### Self-report measure

Participants were asked to select which statement best described them from the following seven options: a) “I strongly prefer thin people to fat people”, b) “I moderately prefer thin people to fat people”, c) “I slightly prefer thin people to fat people”, d) “I prefer thin people and fat people equally”, e) “I slightly prefer fat people to thin people”, f) “I moderately prefer fat people to thin people”, g) “I strongly prefer fat people to thin people”. These were coded as scores from -3 to +3 with more positive scores indicating stronger preferences for thin people over overweight people.

### Data analyses

To investigate the relationship between obesity and weight bias at the individual level, we conducted two linear regressions. In both regressions we used the individual Body Mass Index (BMI) scores as predictor whereas the explicit and implicit individual weight bias were used separately as dependent variables in the first and second regression respectively. The individual BMI was obtained by converting heights and weights [40] self-reported by the participants in the weight questionnaire at the Project Implicit website.

At the national level, we computed national estimates of implicit and explicit weight bias by averaging the individual scores for each nation from the Project Implicit sample to serve as dependent variables. Also, we had three possible national obesity indicators (national BMI, national percentage of overweight and underweight people) to serve as predictors. In order to protect against the possibility of alpha inflation by selective reporting, we conducted and report a sequence of three regressions using each indicator of obesity. The sequence of regressions examined the robustness of the relationship between weight bias and obesity across nations by (1) testing that relationship on its own, (2) testing whether the relationship persisted after removing outliers detected in robustness analyses (see section Tests of Robustness in the Information), and (3) testing whether the relationship persisted after adding 4 nation-level covariates to the regression model indicating health, economic or cognitive factors. Specifically, we included the life expectancy at birth as a heath indicator of national differences in public health, medical care and diet; the Gross Domestic Product (GDP) per capita and health expenditure per capita as economic national indicators of wealth and total expenditure on health respectively; and finally the Intelligence Quotient (IQ) as a cognitive factor of national differences in cognitive and intellectual abilities. Nation-level covariates were obtained from public databases (see Section Data obtained from public databases in the Information).

A detailed explanation of the rationale behind our choice of covariates is provided on page 5 of the Supporting Information. The national weight indicators were obtained from different sources (see section Data obtained from public databases in the Information). Nations were included in the regression models depending on the availability of national weight indicators (Table S2 in File S1). We considered 67 countries in the regression with national BMI, 49 countries in the regression with national percentages of underweight people and 59 countries in the regression with national percentages of overweight people. Given the variability of sample sizes across nations (N=104-226,613), regression analyses were weighted so that the more reliable estimates from larger samples carried more weight than those from smaller samples [41].. The SPSS code that we used for the weighting is provided in the Information. Note that very similar results are observed using unweighted analyses (Table S3, S4 and S5 in File S1) and with an alternative weighting strategy (Table S6 in File S1).

## Results

Overall, the sample showed a preference for thin people over overweight people both with explicit measures (Explicit mean = 1.00, SD = 1.09; t(311,759) = 514.14, *P* < 0.0001, Cohen’s d = 0.92) and with the IAT (IAT mean= 0.43, SD = 0.42; t(303,818) = 556.69, *P* < 0.0001, Cohen’s d = 1.02). Explicit and implicit preferences were positively correlated (*r* = 0.21, *P* < 0.0001). Moreover, without exception, each of the 71 nations showed explicit and implicit weight bias favoring thin people over overweight people, with considerable variability across nations (Table S1 in File S1).

**Figure 1:**
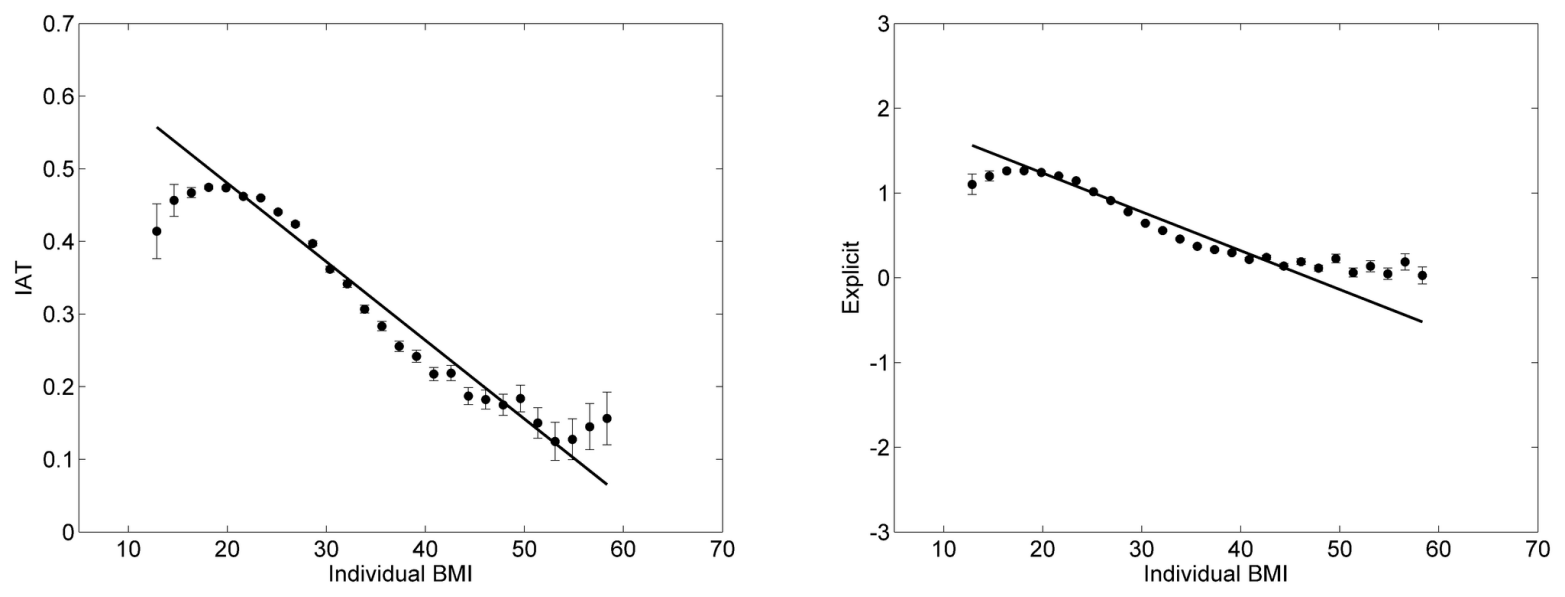
Scatter plots of relations of implicit (IAT) and explicit weight bias with BMI at the individual level. Note. Each point in the plots represents the average preference of participants as a function of their BMI. The weight bias scores ranges from +2 to -2 for the IAT and from +3 to -3 for the explicit, with 0 indicating no relative preference between thin people over overweight people. More positive scores indicate a preference for thin people over overweight people, while more negative scores indicate a preference for overweight people over thin people. Vertical bars signify standard error. Data for participants with BMI greater than 60 were not included in the plot (0.15%). The regression line was computed on the original and not on the average data.

At the individual level, across all participants in the entire sample, BMI scores were negatively related with explicit (β = -0.26, t(302,737) = -149.91, *P* < 0.0001) and implicit (β = -0.16, *t*(291,354) = -87.52, *P* < 0.0001) weight bias (Figure 1). Higher BMI scores were associated with weaker explicit and implicit negativity toward overweight people compared to thin people. However, overweight and obese people still showed explicit and implicit preferences for thin people compared to overweight people. BMI scores were negatively – though not always significantly – correlated with both explicit (r = -0.26, P < 0.0001; mean r = -0.15) and implicit (r = -0.16, P < 0.0001; mean r = -0.13) weight bias in every nation except Bolivia and Vietnam implicitly, and Lithuania and Albania explicitly (Table S7 in File S1).

**Figure 2:**
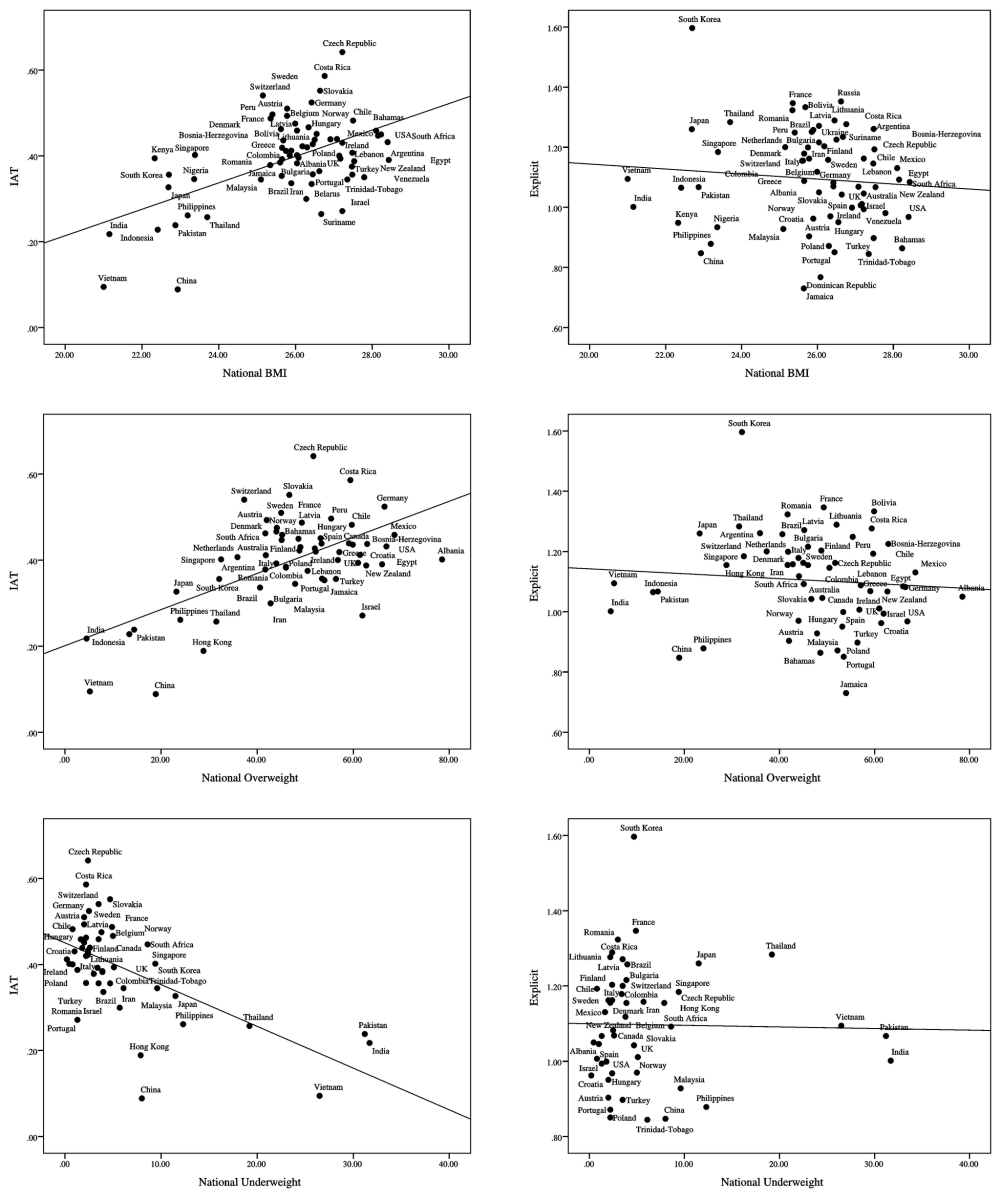
Scatter plots of relations of implicit (IAT) and explicit weight bias as a function of three weight indicators (BMI, percentage of overweight and underweight people) at the national level. Note. The weight bias scores ranges from +2 to -2 for the IAT and from +3 to -3 for the explicit, with 0 indicating no relative preference between thin people over overweight people. More positive scores indicate a preference for thin people over overweight people, while more negative scores indicate a preference for overweight people over thin people.

An opposite relationship between obesity and implicit weight bias was found at the national level, for all three of the operationalizations of obesity (BMI, national percentage of people underweight, national percentage of people overweight). Results of the regressions showed that greater obesity in a nation was associated with stronger implicit negativity toward overweight people compared to thin people [national BMI: β = 0.54, *t*(66) = 5.15, *P* < 0.0001; national percentage of underweight people: β = -0.60, *t*(48) = -5.08, *P* < 0.0001; national percentage of overweight people: β = 0.60, *t*(58) = 5.71, *P* < 0.0001; Figure 2]. Demonstrating robustness, this relationship persisted after removing outliers, [BMI: β= 0.43, *t*(64) = 3.81, *P* < 0.0001; national percentage of underweight people: β = -0.49, *t*(46) = -3.76, *P* < 0.0001; national percentage of overweight people: β = 0.51, *t*(56) = 4.42, *P* < 0.0001] and after including 4 nation-level covariates to the models [BMI: β = 0.36, *t*(64) = 3.04, *P* < 0.01; national percentage of underweight people: β = -0.45, *t*(46) = -3.13, *P* < 0.01; national percentage of overweight people: β = 0.51, *t*(56) = 4.31, *P* < 0.0001]. Moreover, the relationship persisted after adding covariates (Table 1). Across three operationalizations of obesity, and three regressions for each examining the robustness of the relationship to outliers and covariates, the relationship between obesity and implicit weight bias across nations was strong and persistent. Further, supporting information shows additional robustness checks indicating that the obesity-weight bias relationship persists with alternative weighting strategies.

**Table 1:** Weighted regression models predicting the implicit (IAT) and explicit weight bias at the national level.

			**IAT**					**Explicit**				
**Model**	**df**	**Parameter**	**R2**	b*	**SE**	**t**	**p-value**	**R2**	b*	**SE**	**t**	**p-value**
**M1**	66	Intercept	0.290	-0.386	0.152	-2.534	0.014	0.017	1.415	0.303	4.673	0.000
		*BMI*		*0.030*	*0.006*	*5.148*	*0.000*		-0.012	0.012	-1.067	0.290
**M1**	64	Intercept	0.187	-0.251	0.172	-1.459	0.150	0.032	1.598	0.348	4.594	0.000
**Drop Outliers**		*BMI*		*0.025*	*0.007*	*3.809*	*0.000*		-0.019	0.013	-1.450	0.152
**M1**	64	Intercept	0.309	-0.115	0.257	-0.448	0.656	0.110	0.708	0.540	1.310	0.195
**Drop Outliers**		*BMI*		*0.021*	*0.007*	*3.042*	*0.003*		-0.010	0.015	-0.712	0.479
**Add 4 Covariates**		Life expectancy		0.000	0.002	-0.143	0.887		-0.001	0.005	-0.199	0.843
		Health exp.		0.000	0.000	0.080	0.937		0.000	0.000	-0.633	0.529
		GDP		0.000	0.000	1.541	0.129		0.000	0.000	-0.274	0.785
		IQ		-0.001	0.002	-0.327	0.745		0.008	0.004	1.886	0.064
**M2**	48	Intercept	0.354	0.449	0.016	27.774	0.000	0.000	1.091	0.029	37.323	0.000
		*Underweight*		*-0.010*	*0.002*	*-5.078*	*0.000*		0.000	0.003	-0.035	0.972
**M2**	46	Intercept	0.239	0.452	0.018	25.590	0.000	0.004	1.083	0.032	33.541	0.000
**Drop Outliers**		*Underweight*		*-0.010*	*0.003*	*-3.762*	*0.000*		0.002	0.005	0.423	0.675
**M2**	46	Intercept	0.332	0.772	0.251	3.074	0.004	0.046	0.698	0.480	1.455	0.153
**Drop Outliers**		*Underweight*		*-0.009*	*0.003*	*-3.132*	*0.003*		0.003	0.006	0.528	0.600
**Add 4 Covariates**		Life expectancy		-0.001	0.004	-0.342	0.734		0.005	0.008	0.628	0.534
		Health exp.		0.000	0.000	0.582	0.564		0.000	0.000	-0.932	0.357
		GDP		0.000	0.000	1.111	0.273		1.759-6	0.000	0.486	0.630
		IQ		-0.003	0.003	-0.865	0.392		0.000	0.007	-0.016	0.987
**M3**	58	Intercept	0.364	0.208	0.035	5.919	0.000	0.014	1.156	0.067	17.295	0.000
		*Overweight*		*0.004*	*0.001*	*5.708*	*0.000*		-0.001	0.001	-0.903	0.370
**M3**	56	Intercept	0.262	0.231	0.041	5.650	0.000	0.031	1.201	0.079	15.287	0.000
**Drop Outliers**		*Overweight*		*0.004*	*0.001*	*4.420*	*0.000*		-0.002	0.002	-1.329	0.189
**M3**	56	Intercept	0.357	0.384	0.199	1.929	0.059	0.072	0.694	0.400	1.735	0.089
**Drop Outliers**		*Overweight*		*0.004*	*0.001*	*4.030*	*0.000*		-0.001	0.002	-0.815	0.419
**Add 4 Covariates**		Life expectancy		-0.003	0.003	-0.761	0.450		0.001	0.007	0.205	0.838
		Health exp.		0.000	0.000	-0.083	0.934		0.000	0.000	-0.749	0.457
		GDP		0.000	0.000	1.576	0.121		0.000	0.000	0.023	0.982
		IQ		0.000	0.003	-0.077	0.939		0.004	0.005	0.841	0.404

^*^ Unstandardized Coefficients

Across models, none of the covariates were significant predictors. However, after removing the health expenditure per capita from the regression models, which was highly correlated with GDP (*r* = 0.87, *P* < 0.0001; Table S8 in File S1), we found that the GDP was a significant predictor of implicit weight preferences in each three regression models with national weight indicators. When, instead, we removed GDP from regression then health expenditure per capita was a significant predictor of implicit weight bias, but only in the regression model with percentage of underweight people (Table S9 in File S1). This result indicated that richer nations showed stronger weight bias but importantly it did not account for the relationship between the weight indicators and implicit preferences. Notably, no statistically significant effect emerged when implicit measures were replaced by explicit measures in all conducted regression analyses (Table 1; Table S9 in File S1).

## Discussion

 We found that (a) each of the 71 nations investigated showed implicit and explicit preferences for thin people compared to overweight people, (b) at the individual level, higher obesity (i.e., increased individual BMI scores) was associated with *weaker* implicit and explicit weight bias but on average overweight people still showed persistent preferences for thin people (c) at the national level, higher obesity (i.e., increased national BMI scores and percentages of overweight people) was associated with *stronger* implicit weight bias whereas no relation was observed with explicit measures, and (d) independent of weight indicators, national wealth was a significant predictor, with richer nations showing stronger implicit preferences for thin people compared to overweight people.

As the effects we found in our study were observed implicitly, but not explicitly, they suggest that the influence of prevalence of obesity on weight bias at the national level may be consciously denied or rejected. Explicit attitudes indicate evaluations that people are able and willing to report while implicit attitudes are inferred with behavioral measures that assess associations that exist in memory [30] and may thus be less influenced by conscious intentions. Indeed, implicit weight attitudes – in addition to being modestly positively related with explicit weight attitudes [17] – can differ in important ways from self-reported evaluations [14–16]. Furthermore, a recent study showed that self-report measures for investigating cross-national differences is weakened by factors compromising the validity of self-report [42]. In particular, the reference-group effect is the tendency for people to respond to subjective self-report items by comparing themselves with standards from their culture [43,44]. This compromises the sensitivity of detecting differences across nations. This is less likely to affect the implicit measures because participants’ evaluations are estimated from behavioral performance, not based on introspective processes that involve such decision-making biases.

 Our results suggest that the degree of negativity toward overweight and obese people at the national level is in sharp contrast to individuals’ social identity factors that promote preferences for oneself and one’s group identities and characteristics. Whereas the relationship between obesity and weight bias was *negative* across individuals, it was *positive* across nations. This is an instance of Simpson’s paradox [45], in which a relation present at one level of analysis (across individuals) is reversed when examined at another level of analysis (across national aggregates of individuals). Unlike some social groups that are evaluated more positively when they are present in greater numbers, the increased prevalence of obesity in a nation does not seem to attenuate negative implicit social attitudes toward overweight people but exaggerates them. As a consequence, for example, an obese person living in the United States (where a relatively high proportion of the population is overweight) is, on average, likely to have an implicit preference for thin people over overweight people that is as strong as a thin person living in India (where a relatively small proportion of the population is overweight).

The mechanism for this effect is not indicated directly by these data and no existing theoretical frameworks anticipate this particular pattern of effects. However, there are some existing theories and areas of research that offer relevant insights. Here, we offer relatively speculative possibilities that will require additional research to clarify the mediating mechanisms of the present results. For example, as noted in the introduction, the greater prevalence of obesity in a nation may increase the negativity toward overweight and obese people by eliciting more frequent national discussion about the health risks and medical costs of obesity, ultimately exacerbating the stigmatization of obesity. Likewise, the high prevalence of obesity in a nation may reinforce the desirability of the thin ideal or of being normal weight by increase prevalence and emphasis on advertisements for diet plans, healthy foods, and gym memberships against the demographic trend of increasing obesity. Further, the association of thinness with social status – health and beauty – may produce even stronger negativity against obesity when this signal of status is rarer.

 An additional speculative explanation of our findings may be provided by “the just world hypothesis” which asserts that people generally get what they deserve as the result of a universal force that restores moral balance [46,47]. Based on this proposal, the bias toward overweight and obese people might represent a “fair” consequence for their negative (i.e. unhealthy) actions. The different weight bias across nations might indicate differences in a just world belief at the national level. That is, countries with stronger beliefs in a just world might show a greater derogation of overweight and obese people.

Potentially supporting this interpretation is the fact that just world beliefs are a function not only of personal experience, but also of societal functionalism (i.e. a country’s structural and societal factors) [48]. Differences, indeed, have been found at the national and cultural level [49,50]. Furnham [50], for instance, found that nations who have more property, wealth and power had stronger just world beliefs, whereas nations who have little or no power and wealth would have unjust world beliefs. However, we did not measure just world beliefs in order to evaluate this possibility in the present data.

Differences in weight bias across countries may be also explained by attribution theory, which declares that people use information to arrive at casual explanations of events [51]. Specifically, according to Heider [52] people can explain behaviors by internal attributions, such as personal or individual features, or by external attributions, such as situational and environmental variables. Several studies showed that overweight and obese people are stigmatized because their weight is perceived to be caused by factors within personal control (e.g., overeating and lack of exercise) than external factors [53]. Societal attributions about the causes of obesity thus seem to contribute significantly to expressions of weight stigma. A speculative application of these findings to our results is that countries with greater negative weight attitudes might present also greater internal attributions of obesity. Indeed, it has been demonstrated that culture affects how people make attributions [54]. Follow-up research could investigate variation in attributions for obesity across nations.

We also observed a separate relationship between national wealth and implicit weight bias. A possible explanation is that the wealth in a nation may alter the meaning of obesity as an indicator of status. In wealthy nations, obesity may be negatively related with wealth perhaps because high-fat foods are cheaper than alternatives [55]. In this context, being overweight may be perceived as being of a lower economic status. In less wealthy nations, obesity may be positively associated with wealth because access to food resources indicate comparatively high economic status. Thus, the different contextualized meaning of obesity may account for the observed relationship between wealth and implicit weight preferences across nations. These explanations suggest that the national context, such as the prevalence of obesity and wealth in a nation, may influence the attitudes toward obesity.

In addition to several novel results the present study has some limitations. Indeed, it is important to point out that while we have offered a directional interpretation – that the prevalence of obesity and wealth influence implicit weight bias – the data themselves are correlational. The data cannot unambiguously support a causal conclusion, and there may be as yet unidentified third variables that are responsible for the observed relationships. A second limitation is that we did not examine the universe of possible covariates to test the robustness of present relationships, and potential explanatory factors. Thus, in addition to the four covariates that we considered it is possible that other variables could still be identified that account for part or most of the observed relationship.

A third limitation of our study is that while the sample is very large, it is not a random selection of the world, or any national population. Selection biases might be present in the sample of people that learned about the web site, volunteered to participate, and selected the weight task. While we cannot conclusively exclude that selection bias is present in our sample, we cannot identify any plausible reason why variation in selection biases across nations would covary with the variables of interest. Nonetheless, replication with other sampling contexts will be particularly useful to increase confidence in the observed relationships.

Taken together, our results point to the subtle influence of national factors in shaping social evaluations. Formation and change of implicit attitudes is not just a matter of influencing intentions; it also requires consideration of the social realities that shape minds without intention.

## Supporting Information

File S1 **Supporting Information: Materials and Methods; References; Tables S1 to S9.**
Table S1. Demographics and descriptive statistics for measures derived from Project Implicit by country. Table S2. Measures derived from other sources by country. Table S3. Unweighted regression models predicting the implicit and explicit weight bias at the national level. Table S4. Unweighted bivariate correlations among predictors of regression models. Table S5. Unweighted regression models predicting the implicit and explicit weight bias at the national level removing GDP or health expenditure. Table S6. Alternative weighted regression models predicting the implicit and explicit weight bias at the national level. In this case, the weighting is given by sample size – 2. Table S7. Correlations between measures from Project Implicit by country. Table S8. Weighted bivariate correlations among predictors of regression models. Table S9. Weighted regression models predicting the implicit (IAT) and explicit weight bias at the national level removing GDP or health expenditure.
(DOC)
