# Supplementary material for: Overweight People Have Low Levels of Implicit Weight Bias, but Overweight Nations Have High Levels of Implicit Weight Bias
Source: PLoS One. 2013 Dec 17;8(12):e83543. doi: 10.1371/journal.pone.0083543 (PMC3866190; doi:10.1371/journal.pone.0083543)
Supplement: File S1 — Supporting Information: Materials and Methods; References; Tables S1 to S9. Table S1. Demographics and descriptive statistics for measures derived from Project Implicit by country. Table S2. Measures derived from other sources by country. Table S3. Unweighted regression models predicting the implicit and explicit weight bias at the national level. Table S4. Unweighted bivariate correlations among predictors of regression models. Table S5. Unweighted regression models predicting the implicit and explicit weight bias at the national level removing GDP or health expenditure. Table S6. Alternative weighted regression models predicting the implicit and explicit weight bias at the national level. In this case, the weighting is given by sample size – 2. Table S7. Correlations between measures from Project Implicit by country. Table S8. Weighted bivariate correlations among predictors of regression models. Table S9. Weighted regression models predicting the implicit (IAT) and explicit weight bias at the national level removing GDP or health expenditure. (DOC) [file pone.0083543.s001.doc]

Supporting Information for

**Overweight people have *low* levels of implicit weight bias, but overweight nations have *high* levels of implicit weight bias**

Maddalena Marini^1,2^, Natarajan Sriram^1^, Konrad Schnabel^3^, Norbert Maliszewski^4^, Thierry Devos^5^, Bo Ekehammar^6^, Reinout Wiers^7^, Cai HuaJian^8^, Mónika Somogyi^9^, Kimihiro Shiomura^10^, Simone Schnall^11^, Félix Neto^12^, Yoav Bar-Anan^13^, Michelangelo Vianello^14^, Alfonso Ayala^15^, Gabriel Dorantes^16^, Jaihyun Park^17^, Selin Kesebir^18^, Antonio Pereira^19^, Bogdan Tulbure^20^, Tuulia Ortner^21^, Irena Stepanikova^22^, Anthony G. Greenwald^23^, Brian A. Nosek^1^

^1^ Department of Psychology, University of Virginia, Charlottesville, VA, USA. ^2^ Department of Communication and Economics, University of Modena and Reggio Emilia, Reggio Emilia, Italy. ^3^ Department of Psychology, University of Potsdam, Potsdam, Germany. ^4^ Department of Psychology, Warsaw University, Warsaw, Poland. ^5^ Department of Psychology, San Diego State University, San Diego, CA, USA. ^6^ Department of Psychology, Stockholm University, Stockholm, Sweden. ^7^ Department of Psychology, University of Amsterdam, Amsterdam, The Netherlands. ^8^ Institute of Psychology, Chinese Academy of Sciences, Beijing, China. ^9^ Department of Psychology, Eötvös Loránd University, Budapest, Hungary. ^10^ Ferris University, Yokohama, Japan. ^11^ Department of Psychology, University of Cambridge, Cambridge, United Kingdom. ^12^ Faculdade de Psicologia e de Ciências da Educação, Universidade do Porto, Porto, Portugal. ^13^ Department of Psychology, Ben-Gurion University of the Negev, Beer Sheva, Israel. ^14^ Department of Applied Psychology, University of Padova, Padova, Italy. ^15^ Instituto Electoral Veracruzano, Xalapa, Mexico. ^16^ Facultad de Psicología, Universidad Autónoma del Estado de Morelos, Cuernavaca, Morelos, México. ^17^ Department of Psychology, Baruch College-City University of New York, New York, NY. ^18^ London Business School, Regent's Park, London, UK ^19^ Brain Institute Federal, University of Rio Grande do Norte, Natal, RN, Brazil. ^20^ Department of Psychology, Transilvania University of Brasov, Brasov, Romania. ^21^ Department of Psychology, Division of Psychological Assessment, University of Salzburg, Salzburg, Austria ^22^ University of South Carolina, Sloan College, Columbia, USA. ^23^ Department of Psychology, University of Washington, Seattle, USA.

This file includes:

Materials and Methods

References

Tables S1 to S9

**Materials and Methods**

**Data analyses**

Following prior practice [1], we first constructed inverse variance weights for both the IAT and explicit data as the inverse of the standard errors. We then log-transformed the IAT and the explicit weights and we averaged them to arrive at a single weighting variable.

Further, we ran the regressions again with an alternate weighting strategy that substantially reduced the weight of the larger samples (dividing by sample size – 2). This alternate weighting strategy replicated the effects with the implicit measures and introduced a significant effect with the explicit measures (Table S6). We conservatively refrain from elaborating on the latter positive result as it is not observed consistently across weighting strategies.

*Regression Analyses*

**Weighted Regressions**

Example of SPSS syntax for weighting

*Weights based on IAT data

COMPUTE IAT_weight = (1/(IAT_se)**2).

COMPUTE log_IAT_weight = LN (1/(IAT_se)**2).

*Weights based on explicit data.

COMPUTE Explicit_weight = (1/(Explicit_se)**2).

COMPUTE log_Explicit_weight = LN (1/(Explicit_se)**2).

*Compute weigths combining IAT and explicit.

*Compute averaged weights. Start by getting means of the above weights to allow computation of new weights with preserved zero points on scales shrunk and stretched so that the weights being averaged have means = 1.0.

COMPUTE DUMMY = 1.

AGGREGATE OUTFILE = * MODE = ADDVARIABLES

/ BREAK = DUMMY

/Mn_IAT Mn_Explicit

= MEAN (log_IAT_weight log_Explicit_weight).

COMPUTE weight= MEAN (log_IAT_weight/(Mn_IAT), log_Explicit_weight/ (Mn_Explicit)).

EXECUTE.

**Unweighted Regressions**

In the unweighted regression models, implicit weight bias was the only dependent variable strongly related to national BMI (β = 0.55, *P* < 0.0001), to national percentage of underweight (β = -0.62, *P* < 0.0001) and overweight people (β = 0.62, *P* < 0.0001). The effects of implicit weight bias remained significant both after removing and after including 4 covariates in the models (Table S3).

Unweighted bivariate correlations among predictors of regression models are shown in Table S4. The effects of unweighted regression with and without the GDP or health expenditure per capita are presented in Table S5.

**Regression Diagnostics for Influential Observations**

In each of three focal unweighted and weighted regressions India and Vietnam emerged as extreme leverage outliers. In the unweighted and weighted regressions India exceeded also on Cook’s D threshold in the regressions of the implicit or explicit data on national percentage of underweight people, and in the regression of the explicit data on national percentage of overweight people. Vietnam exceeded also on Cook’s D threshold in the regressions of the implicit weight bias on each of three predictors. In addition, in the weighted regressions, India, again, exceeded also in the regression of the explicit data on national BMI.

Example of the SPSS code we employed for the outlier investigation, adapted to UCLA [2] guidelines for regression diagnostics.

*Weighted regression diagnostic of IAT on national BMI

REGRESSION

/MISSING LISTWISE

/REGWGT = weight

/STATISTICS COEFF OUTS R ANOVA

/CRITERIA = PIN(.05) POUT(.10)

/NOORIGIN

/DEPENDENT IAT

/METHOD = ENTER National BMI

/RESIDUALS = ID (country) OUTLIERS (SDRESID, LEVER, COOK).

**Rationale behind our choice of covariates**

In our study, we included three categories of covariates that might account for the relationship between obesity and weight bias at the national level: (1) **cognitive level**, (2) **health** and (3) **economic** status of a country. In the following we provide the rationale for our choice of these three factors together with relevant literature.

**Cognitive level of a country** – To investigate the effect of the cognitive level of a country on the relationship between obesity and weight bias, we included in the regression models the Intelligence Quotient (IQ). From a cognitive point of view, the stereotyping process can be defined as a categorization process, in which information are organized based on their features in order to identify, recall, predict and react to them easily [3]. Since the cognitive nature and function of stereotypes, we decided thus to include in our analyses the IQ as an index of the cognitive abilities to test the influence of this factor on the stereotyping process toward obese and overweight people.

**Health level of a country** – To investigate the effect of the health level of a country on the relationship between obesity and weight bias, we included in the regression models the life expectancy at birth. We decided to include the life expectancy at birth as an indicator of the health level of a country because this index reflects differences in public health, medical care, diet and life style [4] and thus it may have an impact on the obesity and its perception.

**Economic status of a country** - To investigate the effects of economic factors on the relationship between obesity and weight bias, we included in the regression models the GDP and health expenditure per capita. The inclusion of GDP per capita was supported by previous studies showing that obesity as an indicator status may have a different meaning based on the wealth of a country. Indeed, in countries with higher levels of development obesity is associated with lower socioeconomic strata [5,6], while people in higher socioeconomic groups are associated to have a healthier diet, characterized by greater consumption of fruit, vegetables, which are more expensive [7–9], and less consumption of fats [10]. In this context, obesity may be considered negatively because it is associated with a lower economic status. Conversely, in countries with lower level of wealth and access to the food resources, the obesity and thus the ability to afford food may produce positive attitudes as an indicator of high economic status.

Similarly, we included the health expenditure per capita as an economic indicator of total expenditure on medical care in a country to investigate whether countries with higher health expenditure, providing more health services addressed to a healthier lifestyle - including nutrition activities, healthy food education and physical activity - may influence weight attitudes by conveying heightened exposure and pressure for thinness. In line with this hypothesis, studies showed that highly affluent contexts in which it also likely the presence of more services - such as gym, weight loss centers and availability of diet food - provide a more constant reminder of the “thin ideal” and a consequent increase of the body dissatisfaction [11,12].

**Tests of robustness**

To test the robustness of our regression analysis between weight bias and the weight indicators at the national level, we subjected our data to a series of increasingly stringent tests. First, we removed two high leverage country outliers (i.e., India and Vietnam) identified by regression diagnostics. In the regression diagnostic, we calculated studentized deleted residuals, centered leverage values and Cook’s D statistics for each of the three primary regression analyses. We considered for the BMI (N=67), percentage of underweight (N=49) and overweight people (N=59) regressions respectively the following thresholds for each index according to UCLA [2] guidelines for regression diagnostics: studentized deleted residuals > |2|; centered leverage values and Cook’s D > 0.06, 0.08, 0.07. In each of three focal unweighted and weighted regressions India and Vietnam emerged as extreme leverage outliers (see SI for SPSS code).

**Data obtained from public databases**

National BMI was obtained from the Global Burden of Metabolic Risk Factors Collaborating Group [13], whereas national percentages of underweight (BMI<18.5) and overweight (BMI>24.9) people were obtained from the World Health Organization [14]. Notably, the national weight indicators from public databases were positively correlated with the respective national estimates obtained from the Project Implicit (national BMI vs. national estimates of BMI: *r=* 0.517, *P* < 0.0001; national percentage of underweight people vs. national estimates of percentage of underweight people: *r* = 0.464 *P* < 0.001; national percentage of overweight people vs. national estimates of percentage of overweight people, *r* = 0.529, *P* < 0.0001). These results indicate that our sample’s distribution of obesity across nations was positively related to other estimates of the distribution of obesity across nations.

Nation-level covariates were obtained from public databases. Life expectancy at birth was obtained from the Central Intelligence Agency’s “World Factbook” [15], GDP per capita was obtained from the Central Intelligence Agency’s “World Factbook” [16], health expenditure per capita was obtained from the World Health Organization [17] and IQ was obtained from a recent study conducted by Rindermann and Thompson [18]. Nations were included depending on the availability of national indicators (Table S2). We considered 65 countries in the regression with national BMI, 47 countries in the regression with national percentages of underweight people and 57 countries in the regression with national percentages of overweight people.

**References**

1. Nosek BA, Smyth FL, Sriram N, Lindner NM, Devos T, et al. (2009) National differences in gender-science stereotypes predict national sex differences in science and math achievement. Proceedings of the National Academy of Sciences of the United States of America 106: 10593–10597.

2. Chen X, Ender P, Mitchell M, Wells C (2003) Regression with SPSS. Available: http://www.ats.ucla.edu/stat/spss/webbooks/reg/default.htm.

3. Tajfel H (1981) Social stereotypes and social groups. In: Turner J, Giles H, editors. Intergroup behavior. Chicago: University of Chicago Press. pp. 144–167.

4. The World Bank Group (2001) Life expectancy. Available: http://www.worldbank.org/depweb/english/modules/social/life/index.html.

5. Sobal J, Stunkard AJ (1989) Socioeconomic status and obesity: a review of the literature. Psychological Bulletin 105: 260–275.

6. McLaren L (2007) Socioeconomic status and obesity. Epidemiologic Reviews 29: 29–48.

7. Travers KD, Cogdon A, McDonald W, Wright C, Anderson B, et al. (1997) Availability and cost of heart healthy dietary changes in Nova Scotia. Journal of the Canadian Dietetic Association 58: 176–183.

8. Drewnowski A, Specter SE (2004) Poverty and obesity: the role of energy density and energy costs. The American Journal of Clinical Nutrition 79: 6–16.

9. Drewnowski A, Darmon N (2005) The economics of obesity: dietary energy density and energy cost. The American Journal of Clinical Nutrition 82: 265S–273S.

10. Power EM (2005) Determinants of healthy eating among low-income Canadians. Canadian Journal of Public Health 96 Suppl 3: S37–42, S42–8.

11. McLaren L, Gauvin L (2002) Neighbourhood level versus individual level correlates of women’s body dissatisfaction: toward a multilevel understanding of the role of affluence. Journal of Epidemiology and Community Health 56: 193–199.

12. McLaren L, Gauvin L (2003) Does the “average size” of women in the neighbourhood influence a woman’s likelihood of body dissatisfaction? Health & Place 9: 327–335.

13. Finucane MM, Stevens GA, Cowan MJ, Danaei G, Lin JK, et al. (2011) National, regional, and global trends in body-mass index since 1980: systematic analysis of health examination surveys and epidemiological studies with 960 country-years and 9·1 million participants. Lancet 377: 557–567.

14. World Health Organization (2011) Global Database on Body Mass Index. Available: http://apps.who.int/bmi/index.jsp and http://country-bmi.findthedata.org. Accessed 19 January 2011.

15. Central Intelligence Agency (2008) The World Factbook. Available: https://www.cia.gov/library/publications/the-world-factbook/fields/2102.html. Accessed 19 January 2011.

16. Central Intelligence Agency (2010) The World Factbook. Available: https://www.cia.gov/library/publications/the-world-factbook/rankorder/2004rank.html. Accessed 19 January 2011.

17. World Health Organization (2009) World Health Statistics. Available: http://www.who.int/whosis/whostat/2009/en/index.html.

18. Rindermann H, Thompson J (2011) Cognitive capitalism: the effect of cognitive ability on wealth, as mediated through scientific achievement and economic freedom. Psychological Science 22: 754–763.

**Table S1. Demographics and descriptive statistics for measures derived from Project Implicit by country.**

| **Country** | **Sessions** | **BMI** | | | **IAT** | | | **Explicit** | | | **Male** | **Age** |
| --- | --- | --- | --- | --- | --- | --- | --- | --- | --- | --- | --- | --- |
|  | **N** | **N** | **Mean** | **SD** | **N** | **Mean** | **SD** | **N** | **Mean** | **SD** | **Percent** | **Mean** |
| **Albania** | 129 | 93 | 24.41 | 6.92 | 90 | 0.40 | 0.42 | 100 | 1.05 | 1.27 | 37.98 | 26.91 |
| **Argentina** | 725 | 632 | 24.17 | 5.11 | 623 | 0.41 | 0.44 | 644 | 1.26 | 1.09 | 45.35 | 29.17 |
| **Australia** | 4,828 | 4,314 | 25.15 | 6.51 | 4,345 | 0.43 | 0.42 | 4,448 | 1.05 | 1.08 | 28.74 | 29.41 |
| **Austria** | 889 | 812 | 23.90 | 5.19 | 792 | 0.49 | 0.41 | 796 | 0.90 | 1.02 | 36.17 | 30.68 |
| **Bahamas** | 112 | 85 | 26.86 | 8.30 | 88 | 0.45 | 0.43 | 88 | 0.86 | 1.33 | 37.84 | 26.03 |
| **Belarus** | 109 | 75 | 23.11 | 3.76 | 81 | 0.33 | 0.44 | 86 | 1.07 | 1.36 | 34.86 | 26.66 |
| **Belgium** | 2,036 | 1,875 | 23.48 | 4.51 | 1,814 | 0.48 | 0.42 | 1,888 | 1.12 | 1.09 | 42.77 | 29.00 |
| **Bolivia** | 122 | 88 | 23.90 | 5.67 | 88 | 0.44 | 0.42 | 96 | 1.33 | 1.12 | 42.15 | 27.45 |
| **Bosnia-Herzegovina** | 134 | 106 | 23.94 | 6.44 | 106 | 0.44 | 0.40 | 111 | 1.23 | 1.17 | 45.80 | 23.89 |
| **Brazil** | 1,547 | 1,302 | 24.25 | 4.87 | 1,377 | 0.34 | 0.44 | 1,441 | 1.26 | 1.15 | 42.92 | 28.13 |
| **Bulgaria** | 200 | 175 | 22.65 | 4.09 | 180 | 0.38 | 0.44 | 176 | 1.22 | 1.04 | 30.30 | 26.25 |
| **Canada** | 14,290 | 13,010 | 24.56 | 5.87 | 12,879 | 0.44 | 0.42 | 13,017 | 1.07 | 1.07 | 28.09 | 27.80 |
| **Chile** | 183 | 156 | 24.14 | 4.46 | 154 | 0.48 | 0.42 | 166 | 1.19 | 1.17 | 36.46 | 28.22 |
| **China** | 4,706 | 4,027 | 22.02 | 5.77 | 3,795 | 0.09 | 0.49 | 4,308 | 0.85 | 1.07 | 38.26 | 23.63 |
| **Colombia** | 417 | 361 | 23.36 | 4.31 | 370 | 0.38 | 0.41 | 368 | 1.15 | 1.14 | 28.40 | 26.84 |
| **Costa Rica** | 104 | 87 | 24.19 | 4.89 | 93 | 0.59 | 0.41 | 94 | 1.28 | 1.13 | 34.95 | 27.40 |
| **Croatia** | 115 | 105 | 23.26 | 4.15 | 100 | 0.41 | 0.43 | 106 | 0.96 | 1.08 | 26.96 | 25.98 |
| **Czech Rep** | 278 | 261 | 22.89 | 3.58 | 255 | 0.64 | 0.41 | 265 | 1.16 | 1.00 | 33.33 | 25.83 |
| **Denmark** | 564 | 501 | 24.12 | 5.28 | 506 | 0.46 | 0.42 | 496 | 1.16 | 1.02 | 34.64 | 28.46 |
| **Dominican Rep** | 148 | 127 | 25.59 | 5.52 | 133 | 0.40 | 0.40 | 129 | 0.77 | 1.14 | 25.68 | 24.55 |
| **Egypt** | 106 | 85 | 25.50 | 6.86 | 92 | 0.39 | 0.41 | 95 | 1.08 | 1.18 | 30.48 | 24.20 |
| **Finland** | 621 | 564 | 23.86 | 5.14 | 543 | 0.42 | 0.43 | 561 | 1.20 | 1.04 | 32.63 | 26.69 |
| **France** | 6,298 | 5,735 | 23.02 | 4.43 | 5,581 | 0.49 | 0.39 | 5,896 | 1.35 | 1.15 | 43.90 | 28.83 |
| **Germany** | 10,340 | 9,579 | 23.87 | 5.24 | 9,444 | 0.52 | 0.42 | 9,649 | 1.08 | 1.03 | 42.08 | 28.81 |
| **Greece** | 251 | 221 | 25.14 | 5.26 | 221 | 0.42 | 0.41 | 228 | 1.09 | 1.13 | 39.52 | 29.66 |
| **Hong Kong** | 329 | 278 | 22.87 | 6.78 | 285 | 0.19 | 0.49 | 297 | 1.15 | 1.03 | 40.73 | 23.99 |
| **Hungary** | 3,768 | 3,402 | 24.24 | 5.32 | 3,308 | 0.45 | 0.43 | 3,181 | 0.95 | 1.03 | 1.79 | 28.92 |
| **India** | 1,531 | 1,356 | 23.86 | 4.38 | 1,305 | 0.22 | 0.44 | 1,377 | 1.00 | 1.15 | 55.09 | 27.81 |
| **Indonesia** | 206 | 167 | 22.26 | 4.97 | 170 | 0.23 | 0.49 | 184 | 1.07 | 1.13 | 27.59 | 22.61 |
| **Iran** | 124 | 109 | 24.30 | 5.63 | 105 | 0.30 | 0.49 | 114 | 1.16 | 1.19 | 38.71 | 28.46 |
| **Ireland** | 988 | 848 | 24.50 | 6.05 | 869 | 0.40 | 0.40 | 896 | 1.01 | 1.08 | 34.45 | 27.98 |
| **Israel** | 2,498 | 2,312 | 23.92 | 5.35 | 2,205 | 0.27 | 0.48 | 2,370 | 0.99 | 1.12 | 29.61 | 27.78 |
| **Italy** | 1,780 | 1,575 | 23.34 | 4.81 | 1,483 | 0.39 | 0.44 | 1,634 | 1.18 | 1.15 | 34.69 | 28.98 |
| **Jamaica** | 219 | 201 | 25.92 | 6.40 | 196 | 0.35 | 0.47 | 200 | 0.73 | 1.05 | 23.39 | 28.53 |
| **Japan** | 3,630 | 3,398 | 21.48 | 3.52 | 3,217 | 0.33 | 0.49 | 3,424 | 1.26 | 1.24 | 42.83 | 26.53 |
| **Kenya** | 118 | 93 | 23.96 | 4.05 | 95 | 0.39 | 0.47 | 97 | 0.95 | 1.33 | 31.03 | 26.19 |
| **Latvia** | 119 | 106 | 21.71 | 2.93 | 108 | 0.46 | 0.39 | 107 | 1.27 | 0.96 | 15.97 | 23.73 |
| **Lebanon** | 108 | 85 | 22.83 | 3.59 | 86 | 0.37 | 0.42 | 96 | 1.15 | 1.24 | 37.04 | 24.92 |
| **Lithuania** | 143 | 127 | 22.67 | 3.66 | 132 | 0.43 | 0.49 | 128 | 1.29 | 1.00 | 32.86 | 25.42 |
| **Malaysia** | 454 | 356 | 22.41 | 4.89 | 388 | 0.34 | 0.47 | 403 | 0.93 | 1.11 | 24.17 | 24.72 |
| **Mexico** | 1,629 | 1,405 | 24.77 | 5.16 | 1,434 | 0.46 | 0.40 | 1,494 | 1.13 | 1.15 | 37.44 | 27.02 |
| **Netherlands** | 5,658 | 5,439 | 23.22 | 3.99 | 5,245 | 0.41 | 0.43 | 5,526 | 1.20 | 0.99 | 33.43 | 29.86 |
| **New Zealand** | 1,055 | 908 | 24.97 | 5.79 | 940 | 0.39 | 0.41 | 954 | 1.07 | 1.06 | 29.82 | 28.48 |
| **Nigeria** | 174 | 141 | 26.38 | 6.71 | 146 | 0.35 | 0.43 | 151 | 0.93 | 1.07 | 35.63 | 27.78 |
| **Norway** | 1,298 | 1,170 | 24.11 | 4.73 | 1,174 | 0.47 | 0.40 | 1,167 | 0.97 | 0.95 | 33.85 | 26.91 |
| **Pakistan** | 171 | 147 | 23.60 | 5.27 | 143 | 0.24 | 0.46 | 149 | 1.07 | 1.23 | 46.75 | 25.61 |
| **Peru** | 196 | 175 | 23.88 | 4.57 | 172 | 0.50 | 0.41 | 181 | 1.25 | 1.07 | 40.00 | 27.97 |
| **Philippines** | 468 | 416 | 23.19 | 5.74 | 404 | 0.26 | 0.46 | 428 | 0.88 | 1.09 | 24.13 | 25.92 |
| **Poland** | 8,227 | 7,810 | 22.76 | 4.26 | 7,475 | 0.42 | 0.42 | 8,132 | 0.87 | 1.02 | 30.81 | 25.51 |
| **Portugal** | 2,143 | 1,800 | 23.08 | 4.04 | 1,945 | 0.36 | 0.42 | 2,081 | 0.85 | 1.05 | 31.44 | 28.62 |
| **Puerto Rico** | 147 | 135 | 25.98 | 7.10 | 128 | 0.38 | 0.47 | 137 | 0.73 | 1.00 | 27.21 | 27.99 |
| **Romania** | 615 | 544 | 22.98 | 4.21 | 538 | 0.38 | 0.43 | 563 | 1.32 | 1.13 | 26.31 | 27.31 |
| **Russia** | 703 | 643 | 22.96 | 4.56 | 612 | 0.36 | 0.43 | 633 | 1.35 | 1.10 | 32.66 | 26.44 |
| **Singapore** | 592 | 505 | 21.58 | 3.67 | 526 | 0.40 | 0.44 | 543 | 1.18 | 1.07 | 28.30 | 23.81 |
| **Slovakia** | 131 | 116 | 23.69 | 5.47 | 120 | 0.55 | 0.45 | 118 | 1.04 | 1.02 | 29.17 | 26.99 |
| **South Africa** | 692 | 537 | 24.85 | 6.16 | 619 | 0.45 | 0.42 | 630 | 1.09 | 1.13 | 29.66 | 29.07 |
| **South Korea** | 1,752 | 1,517 | 21.74 | 4.34 | 1,499 | 0.36 | 0.44 | 1,641 | 1.60 | 1.07 | 30.09 | 24.34 |
| **Spain** | 1,084 | 921 | 23.28 | 4.39 | 939 | 0.44 | 0.40 | 969 | 1.00 | 1.06 | 35.38 | 28.30 |
| **Suriname** | 178 | 164 | 23.18 | 4.24 | 167 | 0.26 | 0.46 | 158 | 1.23 | 1.23 | 35.43 | 28.61 |
| **Sweden** | 6,201 | 5,623 | 23.69 | 4.35 | 5,440 | 0.51 | 0.41 | 5,442 | 1.16 | 1.03 | 39.86 | 31.95 |
| **Switzerland** | 869 | 806 | 22.80 | 4.16 | 787 | 0.54 | 0.41 | 804 | 1.20 | 1.07 | 37.78 | 29.48 |
| **Taiwan** | 617 | 493 | 21.96 | 4.67 | 512 | 0.30 | 0.48 | 576 | 1.10 | 1.00 | 30.51 | 24.11 |
| **Thailand** | 138 | 111 | 21.88 | 4.78 | 122 | 0.26 | 0.43 | 127 | 1.28 | 1.13 | 25.00 | 24.86 |
| **Trinidad-Tobago** | 167 | 152 | 25.99 | 7.22 | 141 | 0.34 | 0.45 | 148 | 0.84 | 1.10 | 23.17 | 29.05 |
| **Turkey** | 1,166 | 981 | 23.26 | 4.47 | 964 | 0.36 | 0.43 | 1,073 | 0.90 | 1.02 | 34.26 | 26.17 |
| **UK** | 9,337 | 8,042 | 24.49 | 5.54 | 8,337 | 0.39 | 0.43 | 8,431 | 1.01 | 1.09 | 39.87 | 31.00 |
| **Ukraine** | 261 | 229 | 23.28 | 6.07 | 238 | 0.40 | 0.42 | 239 | 1.25 | 1.12 | 22.66 | 25.31 |
| **USA** | 226,613 | 212,166 | 25.53 | 6.63 | 204,850 | 0.43 | 0.41 | 208,979 | 0.97 | 1.09 | 26.87 | 27.57 |
| **USMOI** | 205 | 189 | 24.86 | 5.78 | 189 | 0.48 | 0.39 | 191 | 0.87 | 1.09 | 24.63 | 24.01 |
| **Venezuela** | 226 | 194 | 24.20 | 5.07 | 195 | 0.35 | 0.44 | 208 | 0.98 | 1.09 | 28.44 | 27.69 |
| **Vietnam** | 141 | 107 | 20.86 | 2.72 | 116 | 0.09 | 0.49 | 127 | 1.09 | 1.26 | 24.29 | 24.22 |
| **Total** | 338,121 | 312,375 | - | - | 303,819 | - | - | 311,760 | - | - | - | - |
| **Mean** | 4,762 | 4,400 | 23.73 | 5.07 | 4,279 | 0.39 | 0.43 | 4,391 | 1.09 | 1.11 | 33.03 | 27.03 |
| **SD** | 26,842 | 25,131 | 1.23 | 1.09 | 24,266 | 0.10 | 0.03 | 24,754 | 0.17 | 0.09 | 7.92 | 2.02 |

Note. Implicit preference was measured by IAT. Higher scores reflect stronger association of concept thin with good and fat with bad than the reversed pairings. Explicit preference was measured by 7-pt likert scales. Higher scores reflect stronger association of thin with good. **For the convenience of analyses and presentation, we list some territories as nations.**

Table S2. Measures derived from other sources by country.

| **Country** | **BMI** | **Underweight** | **Overweight** | **Life Expectancy** | **Health Expenditure*** | **GDP*** | **IQ** |
| --- | --- | --- | --- | --- | --- | --- | --- |
|  | **Mean** | **Percentage** | **Percentage** | **Mean** | **Mean** | **Mean** | **Mean** |
| **Albania** | 26.04 | 0.45 | 78.48 | 77.96 | 381 | 8,000 | 84.65 |
| **Argentina** | 27.48 | n.a. | 35.90 | 76.56 | 1,205 | 15,000 | 89.17 |
| **Australia** | 27.22 | 1.00 | 49.00 | 81.63 | 3,119 | 41,300 | 100.11 |
| **Austria** | 25.78 | 2.00 | 42.00 | 79.50 | 3,608 | 40,300 | 99.92 |
| **Bahamas** | 28.23 | n.a. | 48.60 | 65.78 | 1,872 | 28,600 | 80.00 |
| **Belarus** | 26.42 | n.a. | n.a. | 70.63 | 623 | 13,400 | 93.09 |
| **Belgium** | 25.99 | 3.80 | 44.10 | 79.22 | 3,236 | 37,900 | 99.47 |
| **Bolivia** | 25.68 | n.a. | 59.90 | 66.89 | 257 | 4,800 | 87.05 |
| **Bosnia-Herzegovina** | 26.50 | n.a. | 62.90 | 78.50 | 616 | 6,600 | 92.27 |
| **Brazil** | 25.89 | 4.00 | 40.60 | 71.99 | 674 | 10,900 | 85.88 |
| **Bulgaria** | 26.04 | 3.90 | 46.00 | 73.09 | 744 | 12,800 | 94.38 |
| **Canada** | 27.08 | 2.60 | 59.10 | 81.23 | 3,673 | 39,600 | 100.85 |
| **Chile** | 27.50 | 0.80 | 59.70 | 77.34 | 174 | 15,500 | 89.13 |
| **China** | 22.93 | 8.00 | 18.90 | 73.47 | 216 | 7,400 | 98.68 |
| **Colombia** | 25.60 | 3.90 | 46.00 | 72.81 | 464 | 9,800 | 83.63 |
| **Costa Rica** | 26.76 | 2.20 | 59.40 | 77.58 | 779 | 11,400 | 86.04 |
| **Croatia** | 25.89 | 0.20 | 61.40 | 75.35 | 1,169 | 17,500 | 97.80 |
| **Czech Rep** | 27.22 | 2.40 | 51.70 | 76.81 | 1,511 | 25,600 | 99.41 |
| **Denmark** | 25.62 | 2.20 | 41.70 | 78.30 | 3,773 | 37,000 | 98.26 |
| **Dominican Rep** | 26.08 | n.a. | n.a. | 73.70 | 379 | 8,600 | 82.01 |
| **Egypt** | 28.43 | n.a. | 66.00 | 72.12 | 320 | 6,200 | 85.53 |
| **Finland** | 26.18 | 2.40 | 48.80 | 78.97 | 2,656 | 35,300 | 102.11 |
| **France** | 25.35 | 4.90 | 49.30 | 80.98 | 3,420 | 33,300 | 98.74 |
| **Germany** | 26.42 | 2.50 | 66.50 | 79.26 | 3,465 | 35,900 | 99.27 |
| **Greece** | 25.65 | n.a. | 57.10 | 79.66 | 2,547 | 30,200 | 96.33 |
| **Hong Kong** | n.a. | 7.88 | 28.84 | 81.66 | n.a. | 45,600 | 104.21 |
| **Hungary** | 26.55 | 2.00 | 53.20 | 73.44 | 1,492 | 19,000 | 98.77 |
| **India** | 21.15 | 31.70 | 4.50 | 69.89 | 86 | 3,400 | 82.01 |
| **Indonesia** | 22.41 | n.a. | 13.40 | 70.76 | 82 | 4,300 | 85.71 |
| **Iran** | 26.28 | 5.70 | 42.80 | 71.14 | 678 | 11,200 | 85.74 |
| **Ireland** | 27.14 | 0.80 | 56.80 | 78.24 | 3,106 | 37,600 | 97.01 |
| **Israel** | 27.22 | 1.30 | 61.90 | 80.73 | 2,034 | 29,500 | 95.04 |
| **Italy** | 25.65 | 3.40 | 44.00 | 80.20 | 2,631 | 30,700 | 98.77 |
| **Jamaica** | 25.64 | n.a. | 54.00 | 73.53 | 307 | 8,400 | 70.94 |
| **Japan** | 22.69 | 11.50 | 23.20 | 82.12 | 2,581 | 34,200 | 103.95 |
| **Kenya** | 22.33 | n.a. | n.a. | 57.86 | 67 | 1,600 | 74.97 |
| **Latvia** | 26.04 | 3.50 | 45.20 | 72.15 | 1,018 | 14,500 | 97.49 |
| **Lebanon** | 27.47 | n.a. | 50.50 | 73.66 | 859 | 14,200 | 84.87 |
| **Lithuania** | 26.45 | 2.40 | 52.00 | 74.90 | 981 | 15,900 | 95.24 |
| **Malaysia** | 25.10 | 9.60 | 47.90 | 73.29 | 544 | 14,700 | 94.79 |
| **Mexico** | 28.10 | 1.65 | 68.58 | 76.06 | 778 | 13,800 | 86.77 |
| **Netherlands** | 25.75 | n.a. | 41.80 | 79.40 | 3,481 | 40,500 | 100.97 |
| **New Zealand** | 27.53 | 1.30 | 62.70 | 80.36 | 2,448 | 28,000 | 100.03 |
| **Nigeria** | 23.36 | n.a. | n.a. | 46.94 | 59 | 2,400 | 76.17 |
| **Norway** | 26.34 | 5.00 | 44.00 | 79.95 | 4,519 | 59,100 | 99.27 |
| **Pakistan** | 22.87 | 31.20 | 14.40 | 64.49 | 47 | 2,600 | 84.03 |
| **Peru** | 25.40 | n.a. | 55.40 | 70.74 | 316 | 9,200 | 82.45 |
| **Philippines** | 23.19 | 12.30 | 24.00 | 71.09 | 120 | 3,500 | 84.88 |
| **Poland** | 26.30 | 2.20 | 52.20 | 76.28 | 919 | 18,800 | 97.73 |
| **Portugal** | 26.45 | 2.20 | 53.50 | 78.21 | 2,199 | 23,000 | 94.12 |
| **Puerto Rico** | n.a. | n.a. | n.a. | 78.53 | n.a. | n.a. | 84.03 |
| **Romania** | 25.34 | 3.00 | 41.70 | 72.45 | 472 | 11,500 | 90.40 |
| **Russia** | 26.62 | n.a. | n.a. | 66.03 | 698 | 15,900 | 97.93 |
| **Singapore** | 23.38 | 9.40 | 32.50 | 81.98 | 1,536 | 62,200 | 104.95 |
| **Slovakia** | 26.64 | 4.70 | 46.70 | 75.40 | 1,279 | 22,200 | 97.93 |
| **South Africa** | 28.15 | 8.60 | 45.10 | 48.98 | 715 | 10,700 | 68.38 |
| **South Korea** | 22.70 | 4.70 | 32.10 | 78.72 | 1,467 | 30,200 | 104.81 |
| **Spain** | 26.91 | 1.80 | 53.40 | 80.05 | 2,466 | 29,500 | 97.80 |
| **Suriname** | 26.67 | n.a. | n.a. | 73.73 | 391 | 9,900 | 89.06 |
| **Sweden** | 25.78 | 2.00 | 45.00 | 80.86 | 3,162 | 39,000 | 99.89 |
| **Switzerland** | 25.15 | 3.50 | 37.30 | 80.85 | 4,179 | 42,900 | 100.73 |
| **Taiwan** | n.a. | n.a. | n.a. | 77.96 | 1,745 | 35,100 | 103.39 |
| **Thailand** | 23.70 | 19.20 | 31.50 | 73.10 | 264 | 8,700 | 91.08 |
| **Trinidad-Tobago** | 27.35 | 6.10 | n.a. | 70.86 | 600 | 22,100 | 89.44 |
| **Turkey** | 27.48 | 3.50 | 56.40 | 71.96 | 584 | 12,300 | 88.99 |
| **UK** | 27.17 | 5.10 | 61.00 | 79.01 | 2,815 | 35,100 | 100.55 |
| **Ukraine** | 25.85 | n.a. | n.a. | 68.25 | 433 | 6,700 | 94.61 |
| **USA** | 28.40 | 2.40 | 66.90 | 78.11 | 6,719 | 47,400 | 98.87 |
| **USMOI** | n.a. | n.a. | n.a. | n.a. | n.a. | n.a. | n.a. |
| **Venezuela** | 27.79 | n.a. | n.a. | 73.61 | 540 | 12,600 | 84.05 |
| **Vietnam** | 21.00 | 26.50 | 5.20 | 71.58 | 151 | 3,100 | 94.09 |
| **Mean** | 25.84 | 5.78 | 46.49 | 74.55 | 1,507 | 21,387 | 92.58 |
| **SD** | 1.74 | 7.16 | 15.55 | 6.66 | 1,404 | 14,711 | 8.39 |

* Health expenditure and Gross Domestic Product (GDP) were per capita.

n.a. = not available.

Note. **For the convenience of analyses and presentation, we list some territories as nations.**

**Table S3. Unweighted regression models predicting the implicit and explicit weight bias at the national level.**

|  |  |  | **IAT** | | | | | **Explicit** | | | | |
| --- | --- | --- | --- | --- | --- | --- | --- | --- | --- | --- | --- | --- |
| **Model** | **df** | **Parameter** | **R2** | **b*** | **SE** | **t** | **p-value** | **R2** | **b*** | **SE** | **t** | **p-value** |
| **M1** | 66 | Intercept | 0.299 | -0.402 | 0.151 | -2.654 | 0.010 | 0.008 | 1.311 | 0.300 | 4.369 | 0.000 |
|  |  | *BMI* |  | *0.031* | *0.006* | *5.268* | *0.000* |  | -0.008 | 0.012 | -0.719 | 0.475 |
| **M1** | 64 | Intercept | 0.186 | -0.245 | 0.171 | -1.436 | 0.156 | 0.017 | 1.458 | 0.348 | 4.193 | 0.000 |
| **Drop Outliers** |  | *BMI* |  | *0.025* | *0.007* | *3.793* | *0.000* |  | -0.014 | 0.013 | -1.040 | 0.302 |
| **M1** | 64 | Intercept | 0.404 | -0.124 | 0.243 | -0.512 | 0.611 | 0.135 | 0.447 | 0.502 | 0.891 | 0.377 |
| **Drop Outliers** |  | *BMI* |  | *0.021* | *0.007* | *3.121* | *0.003* |  | -0.006 | 0.014 | -0.397 | 0.693 |
| **Add 4 Covariates** |  | Life expectancy |  | 0.000 | 0.002 | -0.201 | 0.842 |  | -0.001 | 0.005 | -0.224 | 0.824 |
|  |  | Health exp. |  | 7.035E-6 | 0.000 | 0.478 | 0.635 |  | -1.689E-5 | 0.000 | -0.556 | 0.580 |
|  |  | GDP |  | 1.791E-6 | 0.000 | 1.131 | 0.263 |  | -2.021E-6 | 0.000 | -0.618 | 0.539 |
|  |  | IQ |  | 0.000 | 0.002 | -0.211 | 0.834 |  | 0.010 | 0.004 | 2.430 | 0.018 |
| **M2** | 48 | Intercept | 0.387 | 0.450 | 0.016 | 27.603 | 0.000 | 0.000 | 1.100 | 0.029 | 38.098 | 0.000 |
|  |  | *Underweight* |  | *-0.010* | *0.002* | *-5.443* | *0.000* |  | 0.000 | 0.003 | 0.135 | 0.893 |
| **M2** | 46 | Intercept | 0.256 | 0.450 | 0.017 | 25.847 | 0.000 | 0.002 | 1.094 | 0.031 | 34.889 | 0.000 |
| **Drop Outliers** |  | *Underweight* |  | *-0.010* | *0.002* | *-3.938* | *0.000* |  | 0.001 | 0.004 | 0.276 | 0.784 |
| **M2** | 46 | Intercept | 0.327 | 0.748 | 0.254 | 2.948 | 0.005 | 0.036 | 0.687 | 0.472 | 1.457 | 0.153 |
| **Drop Outliers** |  | *Underweight* |  | *-0.009* | *0.003* | *-3.278* | *0.002* |  | 0.002 | 0.005 | 0.400 | 0.691 |
| **Add 4 Covariates** |  | Life expectancy |  | -0.002 | 0.004 | -0.388 | 0.700 |  | 0.004 | 0.008 | 0.564 | 0.576 |
|  |  | Health exp. |  | 1.315E-5 | 0.000 | 0.792 | 0.433 |  | -2.406E-5 | 0.000 | -0.779 | 0.440 |
|  |  | GDP |  | 1.405E-7 | 0.000 | 0.729 | 0.470 |  | 9.931E-7 | 0.000 | 0.277 | 0.783 |
|  |  | IQ |  | -0.002 | 0.003 | -0.732 | 0.469 |  | 0.001 | 0.006 | 0.140 | 0.889 |
| **M3** | 58 | Intercept | 0.383 | 0.210 | 0.035 | 5.814 | 0.000 | 0.007 | 1.143 | 0.065 | 17.615 | 0.000 |
|  |  | *Overweight* |  | *0.004* | *0.001* | *5.946* | *0.000* |  | -0.001 | -0.001 | -0.617 | 0.540 |
| **M3** | 56 | Intercept | 0.27 | 0.229 | 0.040 | 5.658 | 0.000 | 0.018 | 1.182 | 0.077 | 15.326 | 0.000 |
| **Drop Outliers** |  | *Overweight* |  | *0.004* | *0.001* | *4.509* | *0.000* |  | -0.002 | 0.002 | -1.014 | 0.315 |
| **M3** | 56 | Intercept | 0.366 | 0.37 | 0.192 | 1.924 | 0.06 | 0.082 | 0.596 | 0.380 | 1.567 | 0.123 |
| **Drop Outliers** |  | *Overweight* |  | *0.004* | *0.001* | *4.354* | *0.000* |  | -0.001 | 0.002 | -0.517 | 0.607 |
| **Add 4 Covariates** |  | Life expectancy |  | -0.003 | 0.003 | -0.974 | 0.335 |  | -6.882E-5 | 0.007 | -0.011 | 0.992 |
|  |  | Health exp. |  | 4.424E-6 | 0.000 | 0.285 | 0.777 |  | -2.329E-5 | 0.000 | -0.758 | 0.452 |
|  |  | GDP |  | 2.073E-6 | 0.000 | 1.237 | 0.222 |  | -7.466E-7 | 0.000 | -0.225 | 0.823 |
|  |  | IQ |  | 0.000 | 0.002 | 0.168 | 0.867 |  | 0.007 | 0.005 | 1.361 | 0.180 |

* Unstandardized Coefficients

**Table S4. Unweighted bivariate correlations among predictors of regression models.**

| **Variable** |  | **(1)** | **(2)** | **(3)** | **(4)** | **(5)** | **(6)** | **(7)** | **(8)** | **(9)** |
| --- | --- | --- | --- | --- | --- | --- | --- | --- | --- | --- |
|  | **Mean** | 0.39 | 1.09 | 25.84 | 5.78 | 46.49 | 74.55 | 1,521.68 | 21,386.96 | 92.58 |
|  | **SD** | 0.10 | 0.17 | 1.74 | 7.16 | 15.55 | 6.66 | 1,399.74 | 14,711.37 | 8.39 |
|  |  |  |  |  |  |  |  |  |  |  |
| **(1) IAT** |  | 1 |  |  |  |  |  |  |  |  |
| **(2) Explicit** |  | 0.15 | 1 |  |  |  |  |  |  |  |
| **(3) BMI** |  | *0.55* | -0.09 | 1 |  |  |  |  |  |  |
| **(4) Underweight** |  | *-0.62* | -0.02 | *-0.76* | 1 |  |  |  |  |  |
| **(5) Overweight** |  | *0.62* | -0.08 | *0.83* | *-0.80* | 1 |  |  |  |  |
| **(6) Life Expectancy** |  | 0.20 | 0.12 | 0.19 | *-0.43* | 0.22 | 1 |  |  |  |
| **(7) Health Expenditure** |  | *0.39* | -0.00 | *0.28* | *-0.39* | 0.22 | *0.58* | 1 |  |  |
| **(8) GDP** |  | *0.35* | 0.04 | 0.23 | *-0.38* | 0.13 | *0.65* | *0.88* | 1 |  |
| **(9) IQ** |  | *0.14* | *0,29* | 0.02 | *-0.32* | -0.01 | *0.72* | *0.62* | *0.72* | 1 |

Note. Coefficients in italics are significant, *P* < 0.05.

Table S5. Unweighted regression models predicting the implicit and explicit weight bias at the national level removing GDP or health expenditure.

|  |  |  | **IAT** | | | | | **Explicit** | | | | |
| --- | --- | --- | --- | --- | --- | --- | --- | --- | --- | --- | --- | --- |
| **Model** | **df** | **Parameter** | **R2** | **b*** | **SE** | **t** | **p-value** | **R2** | **b*** | **SE** | **t** | **p-value** |
| **M1** | 64 | Intercept | 0.298 | -0.146 | 0.237 | -0.615 | 0.541 | 0.130 | 0.498 | 0.490 | 1.017 | 0.313 |
| **Drop Outliers** |  | *BMI* |  | *0.022* | *0.007* | *3.314* | *0.002* |  | -0.007 | 0.014 | -0.527 | 0.600 |
| **Add 3 Covariates** |  | Life expectancy |  | 0.000 | 0.002 | -0.206 | 0.838 |  | -0.001 | 0.005 | -0.221 | 0.826 |
| **(No Health Expenditure)** |  | *GDP* |  | *2.377E-6* | *0.000* | *2.386* | *0.020* |  | -3.427E-6 | 0.000 | -1.666 | 0.101 |
|  |  | *IQ* |  | 0.000 | 0.002 | -0.196 | 0.845 |  | *0.010* | *0.004* | *2.426* | *0.018* |
| **M2** | 46 | Intercept | 0.317 | 0.758 | 0.252 | 3.005 | 0.004 | 0.022 | 0.669 | 0.469 | 1.427 | 0.161 |
| **Drop Outliers** |  | *Underweight* |  | *-0.009* | *0.003* | *-3.487* | *0.001* |  | 0.003 | 0.005 | 0.549 | 0.586 |
| **Add 3 Covariates** |  | Life expectancy |  | -0.002 | 0.004 | -0.386 | 0.701 |  | 0.004 | 0.008 | 0.564 | 0.576 |
| **(No Health Expenditure)** |  | GDP |  | 2.523E-7 | 0.000 | 1.932 | 0.060 |  | -1.051E-6 | 0.000 | -0.433 | 0.667 |
|  |  | IQ |  | -0.003 | 0.003 | -0.776 | 0.442 |  | 0.001 | 0.006 | 0.180 | 0.858 |
| **M3** | 56 | Intercept | 0.365 | 0.366 | 0.190 | 1.925 | 0.060 | 0.072 | 0.617 | 0.378 | 1.632 | 0.109 |
| **Drop Outliers** |  | *Overweight* |  | *0.004* | *0.001* | *4.635* | *0.000* |  | -0.001 | 0.002 | -0.748 | 0.458 |
| **Add 3 Covariates** |  | Life expectancy |  | -0.003 | 0.003 | -0.998 | 0.323 |  | 0.000 | 0.006 | 0.025 | 0.980 |
| **(No Health Expenditure)** |  | *GDP* |  | *2.443E-6* | *0.000* | *2.327* | *0.024* |  | -2.693E-7 | 0.000 | -1.291 | 0.202 |
|  |  | IQ |  | 0.000 | 0.002 | 0.184 | 0.855 |  | 0.006 | 0.005 | 1.332 | 0.189 |
| **M1** | 64 | Intercept | 0.286 | -0.181 | 0.238 | -0.757 | 0.452 | 0.129 | 0.510 | 0.488 | 1.045 | 0.300 |
| **Drop Outliers** |  | *BMI* |  | *0.021* | *0.007* | *3.066* | *0.003* |  | -0.005 | 0.014 | -0.372 | 0.711 |
| **Add 3 Covariates** |  | Life expectancy |  | 0.000 | 0.002 | -0.066 | 0.948 |  | -0.001 | 0.005 | -0.302 | 0.764 |
| **(No GDP)** |  | *Health exp.* |  | *1.992E-5* | *0.000* | *2.133* | *0.037* |  | -3.143E-5 | 0.000 | -1.643 | 0.106 |
|  |  | *IQ* |  | 0.000 | 0.002 | 0.120 | 0.905 |  | *0.009* | *0.004* | *2.363* | *0.021* |
| **M2** | 46 | Intercept | 0.319 | 0.665 | 0.226 | 2.948 | 0.005 | 0.034 | 0.629 | 0.417 | 1.508 | 0.139 |
| **Drop Outliers** |  | *Underweight* |  | *-0.009* | *0.003* | *-3.245* | *0.002* |  | 0.002 | 0.005 | 0.430 | 0.669 |
| **Add 3 Covariates** |  | Life expectancy |  | -0.001 | 0.004 | -0.321 | 0.750 |  | 0.005 | 0.008 | 0.600 | 0.551 |
| **(No GDP)** |  | Health exp. |  | 2.203E-5 | 0.000 | 1.959 | 0.057 |  | -1.778E-5 | 0.000 | -0.856 | 0.397 |
|  |  | IQ |  | -0.002 | 0.003 | -0.521 | 0.605 |  | 0.001 | 0.006 | 0.249 | 0.805 |
| **M3** | 56 | Intercept | 0.347 | 0.289 | 0.182 | 1.590 | 0.118 | 0.081 | 0.625 | 0.354 | 1.765 | 0.083 |
| **Drop Outliers** |  | *Overweight* |  | *0.004* | *0.001* | *4.176* | *0.000* |  | -0.001 | 0.002 | -0.488 | 0.627 |
| **Add 3 Covariates** |  | Life expectancy |  | -0.003 | 0.003 | -0.795 | 0.430 |  | 0.000 | 0.006 | -0.045 | 0.964 |
| **(No GDP)** |  | Health exp. |  | 1.932E-5 | 0.000 | 1.958 | 0.056 |  | -2.865E-5 | 0.000 | -1.489 | 0.142 |
|  |  | IQ |  | 0.001 | 0.002 | 0.475 | 0.637 |  | 0.006 | 0.005 | 1.359 | 0.180 |

* Unstandardized Coefficients

**Table S6. Alternative weighted regression models predicting the implicit and explicit weight bias at the national level. In this case, the weighting is given by sample size – 2.**

|  |  |  | **IAT** | | | | | **Explicit** | | | | |
| --- | --- | --- | --- | --- | --- | --- | --- | --- | --- | --- | --- | --- |
| **Model** | **df** | **Parameter** | **R2** | **b*** | **SE** | **t** | **p-value** | **R2** | **b*** | **SE** | **t** | **p-value** |
| **M1** | 66 | Intercept | 0.186 | -0.460 | 0.123 | -0.373 | 0.711 | 0.307 | 2.189 | 0.220 | 9.969 | 0.000 |
|  |  | *BMI* |  | *0.017* | *0.004* | *3.856* | *0.000* |  | *-0.043* | *0.008* | *-5.372* | *0.000* |
| **M1** | 64 | Intercept | 0.141 | 0.010 | 0.130 | 0.077 | 0.939 | 0.343 | 2.324 | 0.229 | 10.130 | 0.000 |
| **Drop Outliers** |  | *BMI* |  | *0.015* | *0.005* | *3.213* | *0.002* |  | *-0.048* | *0.008* | *-5.731* | *0.000* |
| **M1** | 64 | Intercept | 0.344 | -0.039 | 0.408 | -0.094 | 0.925 | 0.356 | 2.589 | 0.870 | 2.976 | 0.004 |
| **Drop Outliers** |  | *BMI* |  | *0.018* | *0.008* | *2.126* | *0.038* |  | *-0.045* | *0.018* | *-2.521* | *0.014* |
| **Add 4 Covariates** |  | Life expectancy |  | 0.004 | 0.004 | 0.942 | 0.350 |  | -0.003 | 0.007 | -0.430 | 0.669 |
|  |  | *Health exp.* |  | *0.000* | *0.000* | *-2.804* | *0.007* |  | 0.000 | 0.000 | -1.180 | 0.243 |
|  |  | *GDP* |  | *0.000* | *0.000* | *3.037* | *0.004* |  | 0.000 | 0.000 | 1.333 | 0.188 |
|  |  | IQ |  | -0.004 | 0.003 | -1.310 | 0.195 |  | -0.002 | 0.006 | -0.277 | 0.783 |
| **M2** | 48 | Intercept | 0.273 | 0.459 | 0.010 | 44.590 | 0.000 | 0.040 | 0.977 | 0.021 | 47.234 | 0.000 |
|  |  | *Underweight* |  | *-0.011* | *0.003* | *-4.201* | *0.000* |  | 0.008 | 0.005 | 1.406 | 0.166 |
| **M2** | 46 | Intercept | 0.258 | 0.474 | 0.013 | 35.312 | 0.000 | 0.100 | 0.947 | 0.027 | 35.011 | 0.000 |
| **Drop Outliers** |  | *Underweight* |  | *-0.017* | *0.004* | *-3.956* | *0.000* |  | *0.019* | *0.009* | *2.235* | *0.030* |
| **M2** | 46 | Intercept | 0.421 | 0.716 | 0.328 | 2.185 | 0.035 | 0.403 | 0.375 | 0.608 | 0.616 | 0.541 |
| **Drop Outliers** |  | *Underweight* |  | *-0.130* | *0.004* | *-3.030* | *0.004* |  | *0.019* | *0.008* | *2.303* | *0.026* |
| **Add 4 Covariates** |  | Life expectancy |  | 0.002 | 0.005 | 0.374 | 0.711 |  | *0.022* | *0.010* | *2.198* | *0.034* |
|  |  | *Health exp.* |  | *0.000* | *0.000* | *-2.156* | *0.037* |  | *0.000* | *0.000* | *-2.009* | *0.051* |
|  |  | *GDP* |  | *0.000* | *0.000* | *2.674* | *0.011* |  | 0.000 | 0.000 | 1.416 | 0.164 |
|  |  | IQ |  | -0.006 | 0.004 | -1.359 | 0.182 |  | -0.012 | 0.008 | -1.579 | 0.122 |
| **M3** | 58 | Intercept | 0.288 | 0.264 | 0.034 | 7.653 | 0.000 | 0.204 | 1.251 | 0.066 | 19.011 | 0.000 |
|  |  | *Overweight* |  | *0.003* | *0.001* | *4.799* | *0.000* |  | *-0.004* | *0.001* | *-3.827* | *0.000* |
| **M3** | 56 | Intercept | 0.238 | 0.275 | 0.037 | 7.355 | 0.000 | 0.233 | 1.287 | 0.071 | 18.245 | 0.000 |
| **Drop Outliers** |  | *Overweight* |  | *0.002* | *0.001* | *4.145* | *0.000* |  | *-0.005* | *0.001* | *-4.083* | *0.000* |
| **M3** | 56 | Intercept | 0.440 | 0.341 | 0.288 | 1.182 | 0.243 | 0.411 | 0.873 | 0.570 | 1.532 | 0.132 |
| **Drop Outliers** |  | *Overweight* |  | *0.003* | *0.001* | *3.780* | *0.000* |  | *-0.004* | *0.002* | *-2.423* | *0.019* |
| **Add 4 Covariates** |  | Life expectancy |  | 0.003 | 0.004 | 0.567 | 0.573 |  | *0.019* | *0.009* | *2.189* | *0.033* |
|  |  | *Health exp.* |  | *0.000* | *0.000* | *-3.263* | *0.002* |  | 0.000 | 0.000 | -1.363 | 0.179 |
|  |  | *GDP* |  | *0.000* | *0.000* | *3.140* | *0.003* |  | 0.000 | 0.000 | 1.535 | 0.131 |
|  |  | IQ |  | -0.004 | 0.003 | -1.244 | 0.219 |  | -0.012 | 0.007 | -1.843 | 0.071 |
| * Unstandardized Coefficients | | |  |  |  |  |  |  |  |  |  |  |

**Table S7. Correlations between measures from Project Implicit by country.**

| **Country** | **IAT-Explicit** | **IAT-BMI** | **Explicit-BMI** |
| --- | --- | --- | --- |
|  | **r** | **r** | **r** |
| **Albania** | *0.25* | -0.12 | 0.01 |
| **Argentina** | *0.26* | *-0.16* | *-0.11* |
| **Australia** | *0.23* | *-0.18* | *-0.26* |
| **Austria** | *0.19* | *-0.15* | *-0.20* |
| **Bahamas** | 0.05 | *-0.32* | *-0.33* |
| **Belarus** | 0.15 | -0.21 | -0.09 |
| **Belgium** | *0.23* | *-0.15* | *-0.24* |
| **Bolivia** | 0.12 | 0.10 | -0.16 |
| **Bosnia-Herzegovina** | 0.03 | -0.15 | *-0.22* |
| **Brazil** | *0.24* | *-0.18* | *-0.17* |
| **Bulgaria** | *0.31* | *-0.16* | *-0.22* |
| **Canada** | *0.19* | *-0.16* | *-0.22* |
| **Chile** | 0.07 | *-0.17* | -0.06 |
| **China** | *0.15* | *-0.08* | -0.03 |
| **Colombia** | *0.20* | -0.06 | *-0.13* |
| **Costa Rica** | 0.01 | -0.06 | -0.07 |
| **Croatia** | 0.10 | *-0.05* | -0.11 |
| **Czech Rep** | *0.24* | -0.13 | -0.09 |
| **Denmark** | *0.21* | *-0.18* | *-0.23* |
| **Dominican Rep** | *0.23* | -0.18 | *-0.28* |
| **Egypt** | 0.13 | -0.18 | *-0.23* |
| **Finland** | *0.16* | *-0.13* | *-0.12* |
| **France** | *0.19* | *-0.12* | *-0.12* |
| **Germany** | *0.21* | *-0.16* | *-0.22* |
| **Greece** | 0.13 | -0.11 | -0.06 |
| **Hong Kong** | *0.13* | -0.04 | -0.11 |
| **Hungary** | *0.19* | *-0.17* | *-0.20* |
| **India** | *0.15* | *-0.10* | *-0.15* |
| **Indonesia** | *0.30* | *-0.29* | -0.13 |
| **Iran** | *0.27* | -0.05 | -0.08 |
| **Ireland** | *0.15* | *-0.10* | -0.06 |
| **Israel** | *0.23* | *-0.20* | *-0.18* |
| **Italy** | *0.27* | *-0.20* | *-0.20* |
| **Jamaica** | *0.20* | -0.07 | -0.13 |
| **Japan** | *0.22* | *-0.14* | *-0.17* |
| **Kenya** | *0.27* | -0.12 | *-0.30* |
| **Latvia** | 0.04 | -0.13 | -0.07 |
| **Lebanon** | *0.26* | -0.06 | -0.06 |
| **Lithuania** | *0.26* | -0.07 | 0.06 |
| **Malaysia** | *0.18* | -0.02 | -0.10 |
| **Mexico** | *0.17* | *-0.13* | *-0.16* |
| **Netherlands** | *0.23* | *-0.17* | *-0.23* |
| **New Zealand** | *0.23* | *-0.10* | *-0.20* |
| **Nigeria** | *0.17* | -0.15 | -0.08 |
| **Norway** | *0.22* | *-0.14* | *-0.18* |
| **Pakistan** | 0.11 | *-0.22* | *-0.17* |
| **Peru** | *0.20* | *-0.21* | -0.07 |
| **Philippines** | *0.21* | -0.08 | *-0.18* |
| **Poland** | *0.18* | *-0.09* | *-0.09* |
| **Portugal** | *0.12* | *-0.10* | *-0.07* |
| **Puerto Rico** | *0.25* | *-0.27* | *-0.22* |
| **Romania** | *0.21* | *-0.10* | *-0.09* |
| **Russia** | *0.11* | *-0.13* | *-0.18* |
| **Singapore** | *0.24* | *-0.12* | -0.06 |
| **Slovakia** | 0.16 | -0.10 | *-0.21* |
| **South Africa** | *0.15* | *-0.15* | *-0.23* |
| **South Korea** | *0.10* | *-0.07* | *-0.07* |
| **Spain** | *0.20* | *-0.16* | *-0.18* |
| **Suriname** | *0.20* | -0.15 | *-0.29* |
| **Sweden** | *0.19* | *-0.10* | *-0.24* |
| **Switzerland** | *0.21* | *-0.08* | *-0.14* |
| **Taiwan** | *0.25* | *-0.13* | -0.07 |
| **Thailand** | 0.12 | *-0.20* | -0.02 |
| **Trinidad-Tobago** | *0.23* | *-0.20* | *-0.21* |
| **Turkey** | *0.14* | *-0.13* | -0.04 |
| **UK** | *0.21* | *-0.14* | *-0.20* |
| **Ukraine** | *0.15* | -0.06 | *-0.20* |
| **USA** | *0.21* | *-0.19* | *-0.29* |
| **USMOI** | 0.04 | -0.10 | *-0.18* |
| **Venezuela** | 0.13 | -0.03 | -0.05 |
| **Vietnam** | 0.08 | 0.06 | -0.04 |
| **Mean** | 0.18 | -0.13 | -0.15 |
| **SD** | 0.07 | 0.07 | 0.08 |

Note. Italic font indicates significant correlation, *P* < 0.005. **For the convenience of analyses and presentation, we list some territories as nations.**

**Table S8. Weighted bivariate correlations among predictors of regression models.**

| **Variable** |  | **(1)** | **(2)** | **(3)** | **(4)** | **(5)** | **(6)** | **(7)** | **(8)** | **(9)** |
| --- | --- | --- | --- | --- | --- | --- | --- | --- | --- | --- |
|  | **Mean** | 0.39 | 1.09 | 25.84 | 5.78 | 46.49 | 74.55 | 1,521.68 | 21,386.96 | 92.58 |
|  | **SD** | 0.10 | 0.17 | 1.74 | 7.16 | 15.55 | 6.66 | 1,399.74 | 14,711.37 | 8.39 |
| **(1) IAT** |  | 1 |  |  |  |  |  |  |  |  |
| **(2) Explicit** |  | 0.16 | 1 |  |  |  |  |  |  |  |
| **(3) BMI** |  | *0.54* | -0.13 | 1 |  |  |  |  |  |  |
| **(4) Underweight** |  | *-0.60* | -0.01 | *-0.74* | 1 |  |  |  |  |  |
| **(5) Overweight** |  | *0.60* | -0.12 | *0.85* | *-0.78* | 1 |  |  |  |  |
| **(6) Life Expectancy** |  | 0.22 | 0.11 | 0.16 | *-0.42* | 0.22 | 1 |  |  |  |
| **(7) Health Expenditure** |  | *0.41* | -0.02 | *0.32* | *-0.38* | *0.29* | *0.57* | 1 |  |  |
| **(8) GDP** |  | *0.38* | 0.05 | *0.25* | *-0.38* | 0.18 | *0.66* | *0.87* | 1 |  |
| **(9) IQ** |  | 0.15 | *0.26* | 0.02 | *-0.33* | 0.03 | *0.74* | *0,61* | *0.73* | 1 |

Note. Coefficients in italic are significant, *P* < 0.005.

Table S9. Weighted regression models predicting the implicit (IAT) and explicit weight bias at the national level removing GDP or health expenditure.

|  |  |  | **IAT** | | | | | **Explicit** | | | | |
| --- | --- | --- | --- | --- | --- | --- | --- | --- | --- | --- | --- | --- |
| **Model** | **df** | **Parameter** | **R2** | **b*** | **SE** | **t** | **p-value** | **R2** | **b*** | **SE** | **t** | **p-value** |
| **M1** | 64 | Intercept | 0.309 | -0.119 | 0.249 | -0.479 | 0.634 | 0.104 | 0.780 | 0.526 | 1.483 | 0.143 |
| **Drop Outliers** |  | *BMI* |  | *0.021* | *0.007* | *3.213* | *0.002* |  | -0.013 | 0.014 | -0.926 | 0.358 |
| **Add 3 Covariates** |  | Life expectancy |  | 0.000 | 0.002 | -0.146 | 0.884 |  | -0.001 | 0.005 | -0.181 | 0.857 |
| **(No Health Expenditure)** |  | *GDP* |  | *0.000* | *0.000* | *2.517* | *0.015* |  | 0.000 | 0.000 | -1.188 | 0.239 |
|  |  | IQ |  | -0.001 | 0.002 | -0.327 | 0.745 |  | 0.008 | 0.004 | 1.877 | 0.065 |
| **M2** | 46 | Intercept | 0.326 | 0.787 | 0.248 | 3.172 | 0.003 | 0.026 | 0.665 | 0.477 | 1.372 | 0.177 |
| **Drop Outliers** |  | *Underweight* |  | *-0.010* | *0.003* | *-3.326* | *0.002* |  | 0.004 | 0.006 | 0.717 | 0.478 |
| **Add 3 Covariates** |  | Life expectancy |  | -0.002 | 0.004 | -0.355 | 0.725 |  | 0.005 | 0.008 | 0.645 | 0.522 |
| **(No Health Expenditure)** |  | *GDP* |  | *0.000* | *0.000* | *2.332* | *0.025* |  | 0.000 | 0.000 | -0.312 | 0.756 |
|  |  | IQ |  | -0.003 | 0.003 | -0.915 | 0.365 |  | 0.000 | 0.007 | 0.049 | 0.961 |
| **M3** | 56 | Intercept | 0.357 | 0.385 | 0.197 | 1.953 | 0.056 | 0.062 | 0.707 | 0.398 | 1.776 | 0.082 |
| **Drop Outliers** |  | *Overweight* |  | *0.004* | *0.001* | *4.273* | *0.000* |  | -0.002 | 0.002 | -1.123 | 0.266 |
| **Add 3 Covariates** |  | Life expectancy |  | -0.003 | 0.003 | -0.764 | 0.448 |  | 0.002 | 0.007 | 0.267 | 0.790 |
| **(No Health Expenditure)** |  | *GDP* |  | *0.000* | *0.000* | *2.400* | *0.020* |  | 0.000 | 0.000 | -0.877 | 0.385 |
|  |  | IQ |  | 0.000 | 0.003 | 0.081 | 0.936 |  | 0.004 | 0.005 | 0.813 | 0.420 |
| **M1** | 64 | Intercept | 0.281 | -0.198 | 0.254 | -0.780 | 0.439 | 0.109 | 0.739 | 0.524 | 1.409 | 0.164 |
| **Drop Outliers** |  | *BMI* |  | *0.020* | *0.007* | *2.916* | *0.005* |  | -0.010 | 0.014 | -0.701 | 0.486 |
| **Add 3 Covariates** |  | Life expectancy |  | 0.000 | 0.002 | 0.084 | 0.934 |  | -0.001 | 0.005 | -0.244 | 0.808 |
| **(No GDP)** |  | Health exp. |  | 0.000 | 0.000 | 1.943 | 0.057 |  | 0.000 | 0.000 | -1.323 | 0.191 |
|  |  | IQ |  | 0.000 | 0.002 | 0.131 | 0.897 |  | 0.008 | 0.004 | 1.903 | 0.062 |
| **M2** | 46 | Intercept | 0.312 | 0.642 | 0.223 | 2.882 | 0.006 | 0.041 | 0.590 | 0.421 | 1.402 | 0.168 |
| **Drop Outliers** |  | *Underweight* |  | *-0.009* | *0.003* | *-3.033* | *0.004* |  | 0.003 | 0.006 | 0.582 | 0.564 |
| **Add 3 Covariates** |  | Life expectancy |  | -0.001 | 0.004 | -0.208 | 0.837 |  | 0.006 | 0.008 | 0.698 | 0.489 |
| **(No GDP)** |  | *Health exp.* |  | *0.000* | *0.000* | *2.105* | *0.041* |  | 0.000 | 0.000 | -0.862 | 0.394 |
|  |  | IQ |  | -0.002 | 0.003 | -0.543 | 0.590 |  | 0.001 | 0.006 | 0.145 | 0.886 |
| **M3** | 56 | Intercept | 0.325 | 0.267 | 0.187 | 1.424 | 0.161 | 0.072 | 0.691 | 0.368 | 1.880 | 0.066 |
| **Drop Outliers** |  | *Overweight* |  | *0.003* | *0.001* | *3.746* | *0.000* |  | -0.001 | 0.002 | -0.842 | 0.403 |
| **Add 3 Covariates** |  | Life expectancy |  | -0.002 | 0.003 | -0.493 | 0.624 |  | 0.001 | 0.007 | 0.215 | 0.831 |
| **(No GDP)** |  | Health exp. |  | 0.000 | 0.000 | 1.756 | 0.085 |  | 0.000 | 0.000 | -1.162 | 0.251 |
|  |  | IQ |  | 0.001 | 0.003 | 0.314 | 0.755 |  | 0.004 | 0.005 | 0.881 | 0.382 |
| * Unstandardized Coefficients | | | | | | | | |  |  |  |  |
